# Relative age as a risk factor for psychiatric diagnoses in children born preterm and to term: a cohort study

**DOI:** 10.1136/bmjpo-2024-003186

**Published:** 2025-04-05

**Authors:** Christine Strand Bachmann, Kari Risnes, Johan Håkon Bjørngaard, Jorun Schei, Sara Marie Nilsen, Kristine Pape

**Affiliations:** 1 Department of Public Health and Nursing, Norwegian University of Science and Technology, Trondheim, Norway; 2 Children’s Clinic, St Olav’s University Hospital, Trondheim, Norway; 3 Department of Clinical and Molecular Medicine, Norwegian University of Science and Technology, Trondheim, Norway; 4 Nord University, Bodo, Norway; 5 Department of Mental Health, Norwegian University of Science and Technology, Trondheim, Norway; 6 Department of Child and Adolescent Psychiatry, St Olav’s University Hospital, Trondheim, Norway

**Keywords:** Neonatology, Epidemiology

## Abstract

**Objective:**

To assess relative age effects (how the youngest children in a school class are at increased risk compared with their older classmates) on healthcare use with psychiatric diagnoses in childhood and adolescence within preterm and term-born boys and girls.

**Design/setting/patients:**

Registry-based cohort study including individuals born in Norway from 1991 to 2012 with follow-up data from the National Patient Registry from 2008 to 2017 when they were aged between 4 and 18 years.

**Exposures:**

Relative age was defined according to birth month and grouped in four 3-month periods. Gestational age at birth (GA week+days) was categorised into preterm (GA 23+0–36+6) and term (GA 37+0–41+6).

**Main outcome measures:**

The presence of psychiatric diagnoses (any diagnosis and specific diagnosis groups according to ICD-10) in data from specialist healthcare contacts at different ages during follow-up was compared between relative age groups in preterm and term born using generalised estimating equation logistic regression analyses.

**Results:**

Of 1 109 411 individuals, 7% were born preterm. Relative age effects for psychiatric diagnosis and specific diagnosis groups were seen for both preterm and term-born boys and girls, with the strongest estimates for any psychiatric diagnosis in the relatively younger preterm girls born in October–December, compared with the relatively older preterm girls born in January–March (OR 1.43 (95% CI 1.25 to 1.63) at ages 4–10 years).

**Conclusions:**

Relative age effects were widely demonstrated for healthcare use with psychiatric diagnoses within term and preterm born, for both girls and boys. The excess risk for those born late in the year added to already existing adversity in children born preterm, emphasising the need for additional consideration related to school and societal structures.

**WHAT IS ALREADY KNOWN ON THIS TOPIC**

- The youngest children in a school class are at increased risk of several mental health outcomes compared with the relatively oldest. Few studies have assessed whether relative age effects may add to mental health risk in children born preterm.

**WHAT THIS STUDY ADDS**

- For both term-born and preterm-born, relative age effects were widely demonstrated for healthcare use related to psychiatric diagnoses. The observed relative age effect in preterm adds to the already known mental health vulnerability in this group.

**HOW THIS STUDY MIGHT AFFECT RESEARCH, PRACTICE OR POLICY**

- Excess mental health burden in preterm children born late in the year emphasises the need for additional consideration related to school and societal structures in preterm born.

## Introduction

 Relative age effect is a term used to describe differences in how the youngest individuals in a school class tend to perform poorer regarding academic, social and health-related outcomes compared with the oldest. Studies have shown pronounced relative age effects concerning academic performance and sports.^1 2^ ADHD (Attention-deficit/ hyperactivity disorder) is another outcome for which the relatively youngest children and adolescents more frequently receive both a diagnosis and medication.^3^,^8^ In the later years, an increasing number of studies have addressed and demonstrated relative age effects for other mental health-related outcomes as well, including emotional and psychosocial well-being, peer problems and even diagnoses like depression and anxiety.^79^,^15^ The results for psychiatric diagnoses have, however, not been conclusive.^16 17^ What does seem clear, though, is that being relatively young brings with it burden in several areas, and that this seems to be related to contextual factors, like school and class organisation.

Many studies have shown that preterm-born children and adolescents, when compared with term-born peers, have increased risk of neuropsychological and social difficulties.^18^,^22^ This may reflect a vulnerability in preterm-born children, which in turn may lead to susceptibility to relative age effects. Such effects for preterm-born children and adolescents have been shown for some educational outcomes.^23^,^25^ We recently demonstrated relative age effects for the use of ADHD medication through late childhood and adolescence in both preterm and term-born children and suggested that the risk imposed by being relatively younger among preterm-born children adds to the already known neuropsychological risks associated with preterm birth.^26^ However, relative age effects related to mental health after preterm birth have only been scarcely studied.

By using national registry data, we aimed to assess the relationship between relative age and psychiatric diagnoses during childhood and adolescence, in girls and boys born preterm and to term.

## Patients and methods

### Patient and public involvement

Our project group collaborates with users (Home - Prematurforeningen) that emphasises needs for more knowledge on the transition from preschool to school for preterm born, however, they have not had any direct influence on analyses or manuscript in this project.

### Study design and follow-up

**Figure 1:**
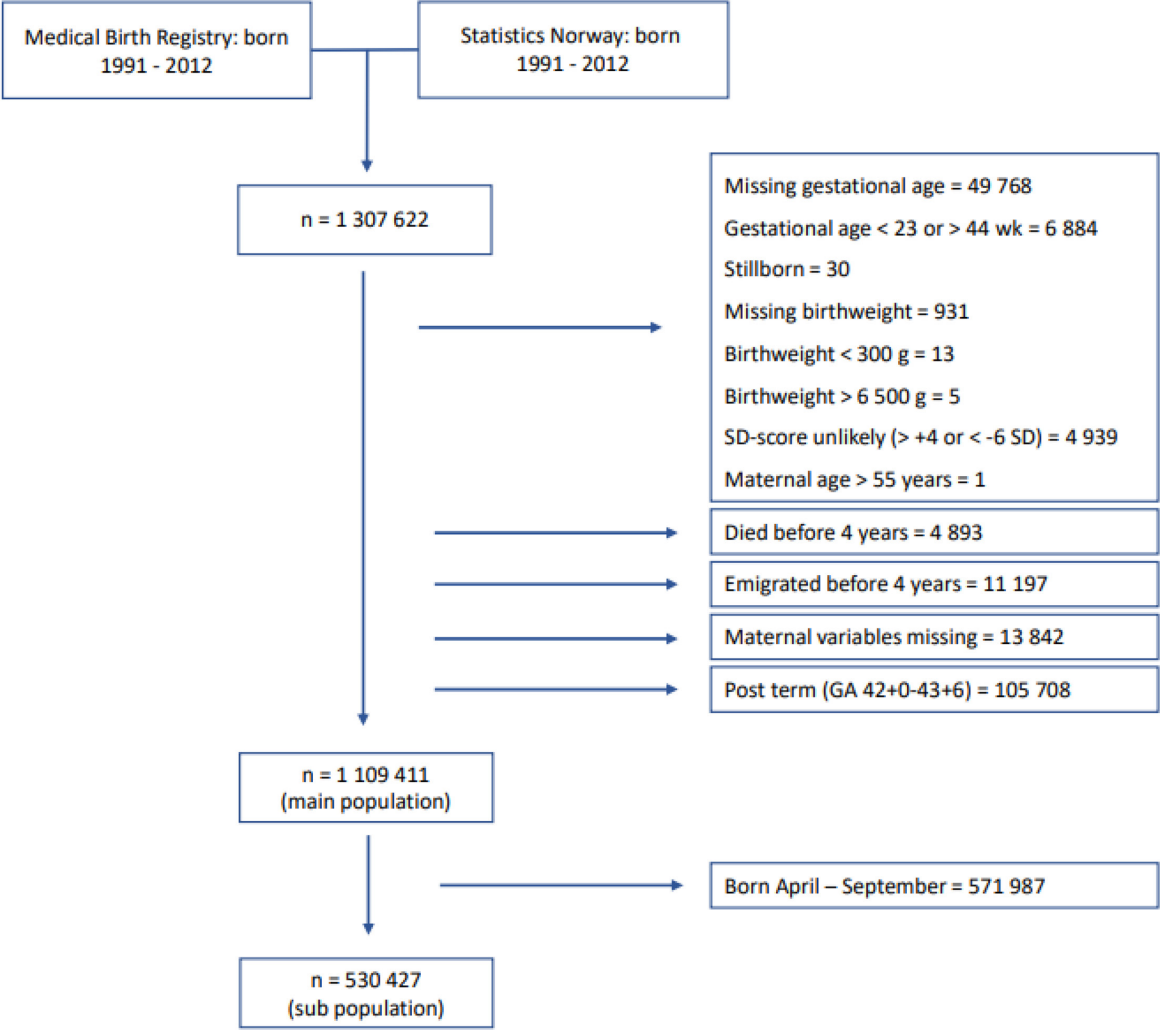
Study population. GA, gestational age.

We used a linkage between the Medical Birth Registry of Norway (MBRN),^27^ the Norwegian Patient Registry (NPR)^28^ and Statistics Norway (SSB)^29^ for this study, using the unique Norwegian identification number to obtain individual level data. The registries provide perinatal and maternal data on all Norwegian residents (MBRN), parental educational data (SSB) and complete information on all contacts and diagnoses received in the somatic and psychiatric specialist healthcare services (NPR). We included all liveborn individuals with registered gestational age (GA, weeks+days) between 23+0 and 42+0 weeks, birth weight between 300 and 6500 g, maternal age under 55 years and registered maternal education. Individuals with unlikely birth weights for GA, that is, more than six SDs below or 4 SDs above the z-score (mean value) according to Marsál *et al*^30^ were excluded (figure 1). Follow-up started in 2008 (first year of NPR) and ended in 2018 (last available data in the current linkage), during which period individuals contributed data for all full years when they were between the age of 4 and 18 years, alive and resident in Norway (online supplemental table S1).

### Exposures

GA was divided into two groups, a preterm group (GA 23+0 to 36+6) and a term group (GA 37+0 to 41+6). An additional categorisation of GA included three subgroups (23+0 to 31+6, 32+0 to 36+6 and 37+0 to 41+6).

Relative age was grouped according to birth month, categorised in four 3-month groups (January–March, April–June, July–September and October–December). In Norway, all children start school in August of the calendar year when they turn 6 years old, and only about 0.6% of Norwegian children delay starting school each year. Individuals born in January–March were defined as the relatively oldest, and those born in October–December as the relatively youngest.

### Outcomes

Outcomes were psychiatric diagnoses from specialist healthcare contacts, defined according to the ICD-10 system,^31^ as registered in NPR. We had access to all ICD-10 codes of mental and behavioural disorders and codes for psychiatric examination and observation, both from paediatric and child and adolescent psychiatric outpatient and inpatient services.

Our primary outcome was any psychiatric diagnosis (F diagnosis). Secondary outcomes included diagnostic groups of F diagnoses commonly seen in childhood and adolescence: ADHD (F90), autism (F84), other neuropsychiatric disorders (F80–83, F88, F89, F95), depression/anxiety (F30–34, F38–39, F40–42, F45, F48, F93) and adjustment/deprivation disorders (F43, F44). Additional outcomes were also assessed: behavioural disorders (F91, F92, F98), relationship disorders (F94), eating disorders (F50), sleep disorders (F51) and psychotic disorders (F20-25). Similar contacts in specialist healthcare regarding psychiatric examination and observation (covered by diagnoses Z00.4 and Z03.2) were included, as were the combination of any Z or F diagnosis, to indicate the total mental health-related burden in the observed children. ICD-10-based definitions of outcomes are presented in online supplemental table S2.

Outcome assessment was done every year during follow-up according to age—from 4 years to 17 years of age—with dichotomous outcome measures reflecting the presence (or not) of the defined diagnostic group for each age interval. Each individual contributed from one to nine repeated measures during follow-up according to availability of data, mainly determined by the year of birth and censoring in the case of emigration or death (online supplemental table S1).

The Norwegian healthcare system permits more than one ICD-10 diagnosis at every visit. Accordingly, each individual may contribute to several of the outcome subgroups at each age interval.

### Covariates

Perinatal and maternal variables collected from MBRN included child’s year of birth (categorical variable), birth weight, sex, multiple birth (singleton vs multiples), congenital birth defects (yes/no), maternal age, parity (0, 1, 2, 3, 4+) relationship status (married/cohabitant vs other) and country of birth (Norway vs other). Parents’ educational level (lower secondary, upper secondary, higher) was collected from SSB. Diagnoses of cerebral palsy (CP) (G80–83) and intellectual disability (F70–79) were collected from NPR. In the analyses, we only included covariates considered as possible confounders in the relationship between relative age and mental health (online supplemental figure S1).

### Statistical analyses

Two main analytical approaches were used to explore and illustrate differences in psychiatric diagnoses from 4 to 17 years according to birth month group (relative age) among term and preterm born. Both were based on repeated annual outcome assessments for each participant analysed using generalised estimating equation (GEE) logistic regression models. The first approach included the full study sample and was chosen to assess how the prevalence of healthcare contact with psychiatric diagnoses developed over the age span for the different birth month groups. For all outcomes, the analyses were performed separately for term and preterm born, boys and girls, and with adjustment for participants’ year of birth and maternal age, parity and education. Follow-up age (at outcome assessment) was included as a categorical variable with 2-year age intervals (4–5, 6–7, 8–9, 10–11, 12–13, 14–15, 16–17), and to allow the development over age to vary between birth month groups, an interaction term was included between birth month group and the age variable. Estimates were used to calculate 1-year prevalences (margins) of each diagnostic outcome for each birth month group at each 2-year age interval, and these were presented graphically. For visibility, only January–March and October–December are shown.

The second approach was chosen to quantify relative age effects and included a subsample of individuals born early in the year (January–March) and late in the year (October–December). For all outcomes, ORs with 95% CIs were estimated to describe relative age effects—the change in odds of having a diagnosis each year for the relatively younger (born October–December) compared with the relatively older (born January–March). Analyses were stratified by GA group and sex and adjusted for participants’ year of birth, maternal age, parity and maternal education. In this analysis, follow-up age (at outcome assessment) was divided into only two periods (4–10 and 11–17 years), in order to provide robust findings but also to allow a statistical evaluation of changes in relative age effects over time (by including an interaction term between birth month group and the age periods). In order to evaluate differences in relative age effects between groups (term vs preterm born, boys vs girls), we performed separate analyses including interaction terms (multiplicative associations) between these variables and birth month groups, at age 4–10 and 11–17 years. The presence of statistical interaction was evaluated systematically and reported whenever present, with p for interaction as a measure of the statistical strength of the group difference.

Additional analyses comparing the late versus early birth month groups (approach two) were performed for selected outcomes (1) in GA subgroups (23+0–31+6, 32+0–36+6 and 37+0–41+6), (2) with additional adjustment for a wider variety of parental background variables (maternal relationship status, country of birth and paternal education in a subsample of participants with available information) and (3) in subsamples excluding individuals diagnosed with CP, intellectual disability, congenital birth defects and from multiple births.

All analyses were done using Stata statistical software V.16.1 (StataCorp).

## Results

### Population

The population consisted of 1 109 411 individuals (figure 1), of which 51.1% were male, and 6.9% born preterm. Table 1 shows that sociodemographic and perinatal characteristics were fairly similarly distributed between the four birth month groups. Online supplemental table S3 displays the subpopulation of 530 427 individuals born in January–March and October–December.

**Table 1:** Descriptives by birth month groups

	January–March*	April–June†	July–September‡	October–December§	All
	n (%)/mean (SD)	n (%)/mean (SD)	n (%)/mean (SD)	n (%)/mean (SD)	n (%)/mean (SD)
Total	275 031 (24.8)	290 621 (26.2)	288 363 (26.0)	255 396 (23.0)	**1 109 411 (100)**
Gender					
Boys	139 996 (50.9)	149 231 (51.4)	147 441 (51.1)	129 829 (50.8)	**566 497 (51.1)**
Girls	135 035 (49.1)	141 390 (48.7)	140 922 (48.9)	125 567 (49.2)	**542 914 (48.9)**
Mean birth weight, g (SD)	3490 (591)	3501 (590)	3498 (585)	3486 (599)	**3494 (591)**
GA					
23+0–36+6 weeks¶	19 147 (7.0)	19 792 (6.8)	19 090 (6.6)	18 594 (7.3)	**76 623 (6.9)**
37+0–41+6 weeks**	255 884 (93.0)	270 829 (93.2)	269 273 (93.4)	236 802 (92.7)	**1 032 788 (93.1)**
Small for gestational age††	11 947 (4.3)	11 893 (4.1)	11 984 (4.2)	11 194 (4.4)	**47 018 (4.2)**
Large for gestational age‡‡	13 443 (4.9)	14 315 (4.9)	13 847 (4.8)	12 336 (4.8)	**53 941 (4.9)**
Mother’s relationship status					
Married/cohabitant	252 548 (91.8)	268 344 (92.3)	266 322 (92.4)	234 930 (92.0)	**1 022 144 (92.1)**
Other	22 483 (8.2)	22 277 (7.7)	22 041 (7.6)	20 466 (8.0)	**87 267 (7.9)**
Multiple births					
Singeltons	264 939 (96.3)	280 416 (96.5)	278 314 (96.5)	146 051 (96.3)	**1 069 720 (96.4)**
Multiple births	10 092 (3.7)	10 205 (3.5)	10 049 (3.5)	9345 (3.7)	**39 691 (3.6)**
Parity					
Primiparae	110 700 (40.3)	114 117 (39.3)	118 421 (41.1)	108 326 (42.4)	**451 564 (40.7)**
Para 1	100 984 (36.7)	108 531 (37.3)	102 899 (35.7)	88 705 (34.7)	**401 119 (36.2)**
Para 2	45 373 (16.5)	49 256 (17.0)	48 264 (16.7)	41 179 (16.1)	**184 072 (16.6)**
Para 3	12 624 (4.6)	13 188 (4.5)	13 149 (4.6)	11 892 (4.7)	**50 853 (4.6)**
Para 4 or more	5350 (2.0)	5529 (1.9)	5630 (2.0)	5294 (2.1)	**21 803 (2.0)**
Maternal mean age, years (SD)	29.1 (5.1)	29.2 (5.1)	29.2 (5.1)	29.2 (5.2)	**29.2 (5.1)**
Maternal education:					
Lower secondary education	63 553 (23.1)	64 601 (22.2)	64 484 (22.4)	60 076 (23.5)	**252 714 (22.8)**
Upper secondary education	101 800 (37.0)	106 383 (36.6)	103 975 (36.1)	92 818 (36.3)	**404 976 (36.5)**
Higher education	109 678 (39.9)	119 637 (41.2)	119 904 (41.6)	102 502 (40.1)	**451 721 (40.7)**
Maternal country of birth					
Norway	227 408 (82.7)	240 552 (82.8)	237 504 (82.4)	207 427 (81.2)	**912 891 (82.3)**
Other	38 022 (13.8)	40 268 (13.9)	41 612 (14.4)	39 765 (15.6)	**159 667 (14.4)**
Congenital birth defects	11 159 (4.1)	11 019 (3.8)	10 686 (3.7)	10 130 (4.0)	**42 994 (3.9)**

Sociodemographic characteristics, perinatal variables and outcomes of the total population.

The bold values display: Total number (percentage).

* Born in January to March.

† Born in April to June.

‡ Born in July to September.

§ Born in October to December.

¶ Gestational age, 23 wk weeks and 0 days to 36 wk weeks and 6 days.

** Gestational age, 37 wk weeks and 0 days to 41 wk weeks and 6 days.

†† Birth weight <2.5th percentile for gestational age.

‡‡ Birth weight >97.5th percentile for gestational age.

%percentGAgestational agennumberSDstandard deviation

### Primary outcome

**Figure 2:**
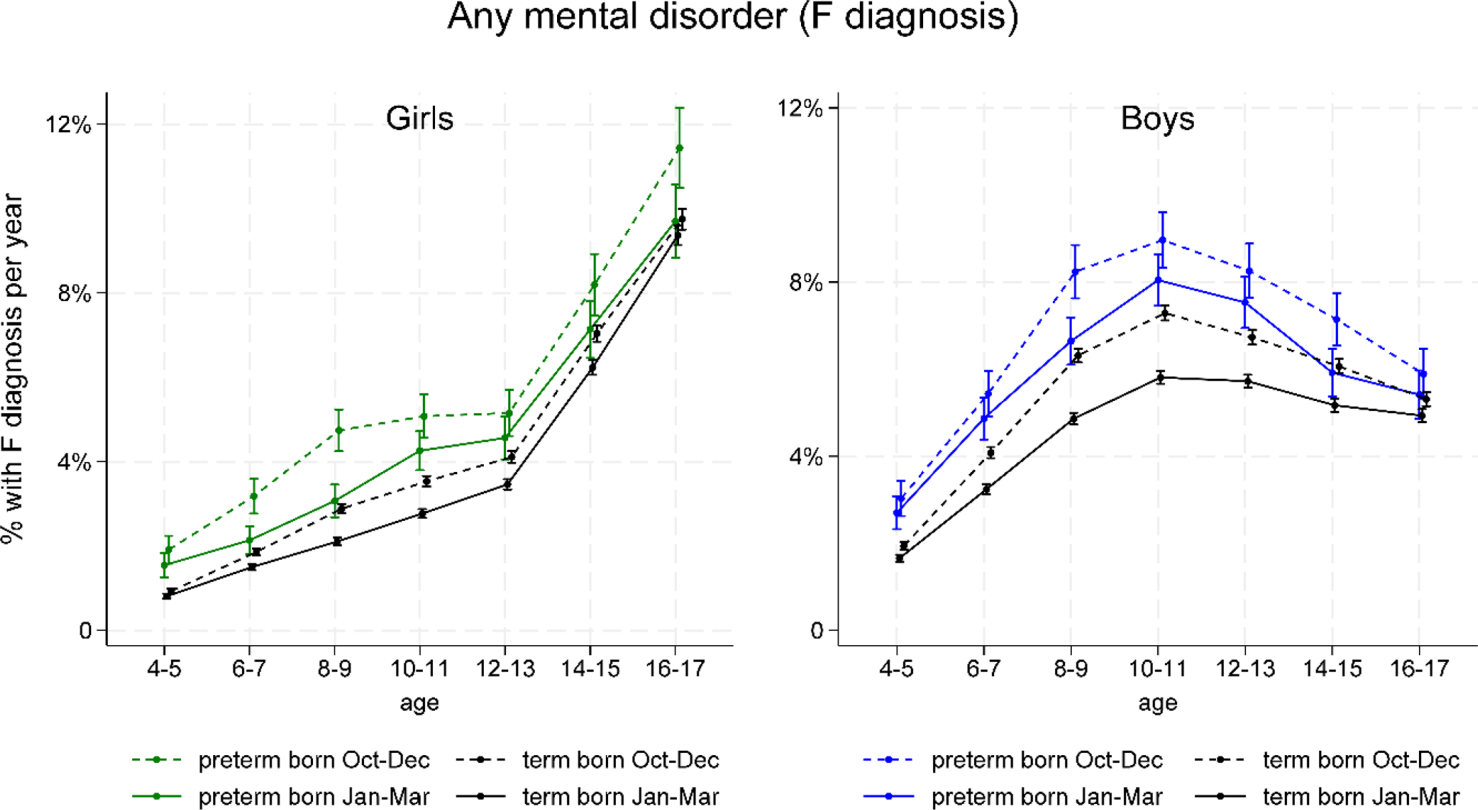
1-year prevalences (in percent) of any F diagnosis from 4 to 17 years according to birth month group (only showing the first and fourth group), estimated from GEE analyses stratified by gestational age and sex, with an interaction term between follow-up age (2-year intervals) and birth month group, and adjusted for birthyear, and maternal age, parity and education. Girls with green and black graphs to the left, boys in blue and black graphs to the right. GEE, generalised estimating equation.

Figure 2 shows how the 1-year prevalence of any registered F diagnosis from age 4–17 years varies between the relative age groups. For boys overall, the prevalence is peaking at about 10 years, while increasing sharply from 12 to 17 years in girls. The group born October–December more often received any F diagnosis among both preterm and term born, boys and girls, compared with the group born in January–March. Those born April–June and July–September were also more often diagnosed than the January–March group, with a dose–response relationship (results not shown). As can be seen in figure 2, children born preterm and late in the year were more often diagnosed than any other group, regardless of sex and across all ages.

Table 2 shows increased ORs of being registered with any F diagnosis for the group born October–December, in comparison with the group born January–March, indicating relative age effects for term and preterm born boys and girls during age periods 4–10 and 11–17 years. Relative age effects were stronger at ages 4–10 years than at ages 11–17 years for preterm girls and term-born boys and girls (p for interaction between age period and birth month group all <0005). Also, relative age effects were stronger for girls than for boys in the preterm group at ages 4–10 years (p for interaction between sex and birth month group 0.022).

**Table 2:** Relative age effects—primary and secondary outcomes

	Preterm				Term			
	Boys		Girls		Boys		Girls	
	OR	95% CI	OR	95% CI	OR	95% CI	OR	95% CI
F diagnosis								
4–10 years	1.15	1.04 to 1.27	1.43	1.25 to 1.63	1.26	1.22 to 1.3	1.27	1.21 to 1.33
11–17 years	1.16	1.06 to 1.27	1.16	1.05 to 1.29	1.17	1.13 to 1.2	1.12	1.09 to 1.16
ADHD								
4–10 years	1.28	1.1 to 1.5	1.53	1.19 to 1.96	1.45	1.37 to 1.53	1.78	1.63 to 1.95
11–17 years	1.25	1.08 to 1.45	1.22	0.98 to 1.51	1.34	1.27 to 1.4	1.49	1.39 to 1.59
Autism								
4–10 years	1.17	0.89 to 1.55	1.17	0.65 to 2.11	1.11	1.01 to 1.21	1.11	0.91 to 1.34
11–17 years	1.39	1.08 to 1.8	0.9	0.58 to 1.41	1.08	1 to 1.17	1.23	1.07 to 1.41
Other neuropsychiatric disorders								
4–10 years	1.05	0.88 to 1.26	1.55	1.18 to 2.02	1.36	1.28 to 1.45	1.27	1.15 to 1.4
11–17 years	1.08	0.92 to 1.27	0.94	0.75 to 1.19	1.2	1.13 to 1.27	1.23	1.14 to 1.33
Depression/anxiety								
4–10 years	1.07	0.81 to 1.4	1.4	1.04 to 1.89	1.12	1.02 to 1.22	1.09	0.98 to 1.2
11–17 years	1.09	0.9 to 1.31	1.17	1.02 to 1.36	1.07	1.02 to 1.13	1.08	1.03 to 1.12
Adjustment/deprivation disorders								
4–10 years	0.77	0.52 to 1.14	1.76	1.14 to 2.72	1.13	1 to 1.27	1.06	0.93 to 1.21
11–17 years	0.89	0.66 to 1.19	1.38	1.11 to 1.7	1.09	1 to 1.19	1.03	0.96 to 1.09

Adjusted ORs (with 95% CI) of annual psychiatric diagnoses form healthcare contacts at ages 4–10 and 11–17 years among the relatively younger preterm and term boys and girls born in October–December, compared with the relatively older groups born in January–March.

ADHDAttention-deficit/ hyperactivity disorder

### Neuropsychiatric disorders

**Figure 3:**
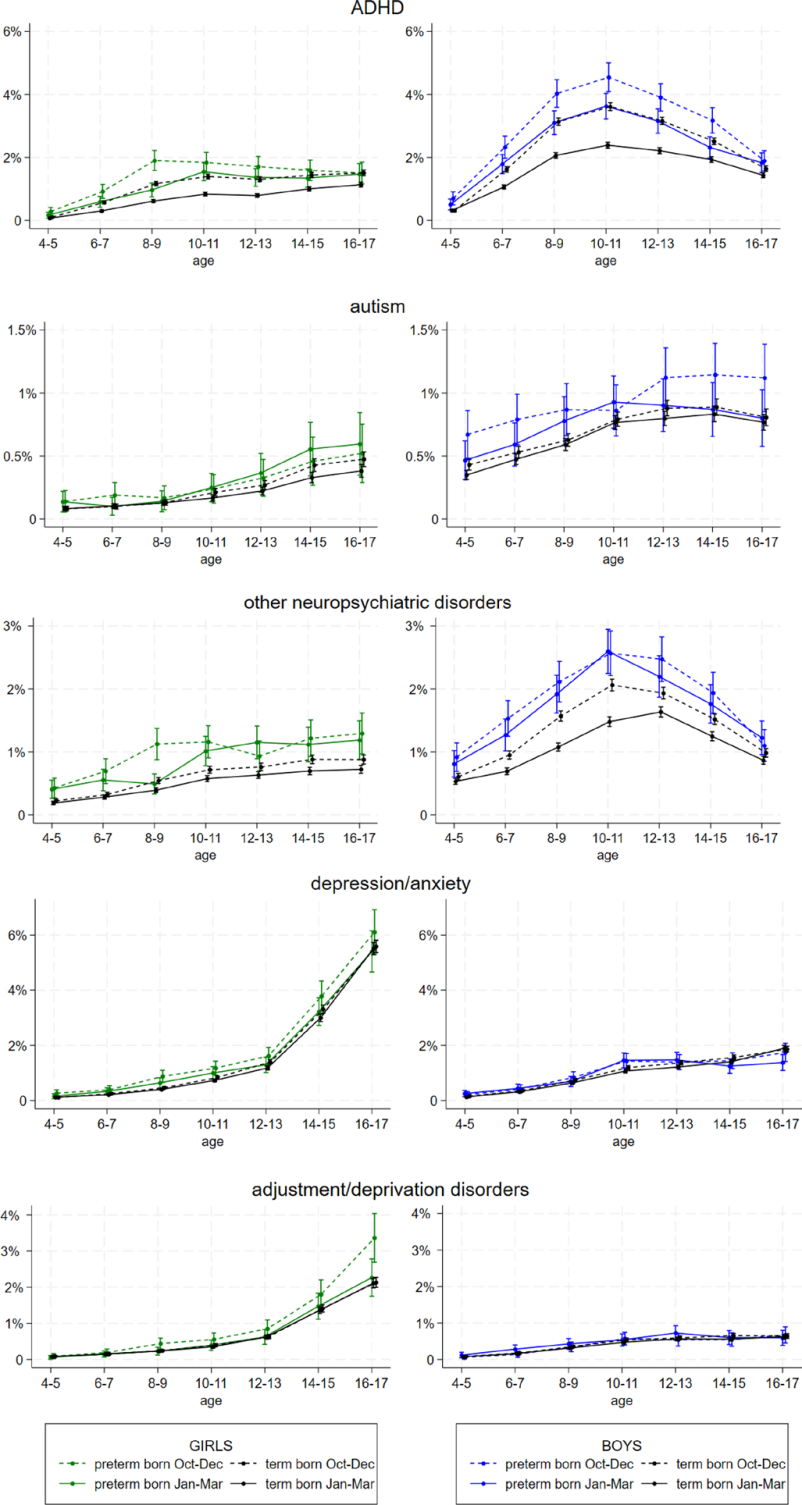
1-year prevalences (in per cent) of ADHD, autism, other neuropsychiatric disorders, anxiety/depression and adjustment/deprivation disorders from 4 to 17 years according to birth month group (only showing the first and fourth group), estimated from GEE analyses stratified by gestational age and sex, with an interaction term between follow-up age (2-year intervals) and birth month group, and adjusted for birth year, maternal age, parity and maternal education. Girls with green and black graphs to the left, boys in blue and black graphs to the right. GEE, generalised estimating equation; ADHD, Attention-deficit/ hyperactivity disorder.

Boys and preterm-born children were more often registered with a neuropsychiatric condition (ADHD, autism and other neuropsychiatric disorders). For ADHD, relative age effects were observed across all ages for all groups (figure 3), with similar patterns for other neuropsychiatric disorders. Preterm born girls born in October–December had an increase in odds of 50% for both ADHD and other neuropsychiatric disorders in ages 4–10 (OR 1.53 (95% CI 1.19 to 1.96) and 1.55 (95% CI 1.18 to 2.02)) compared with their peers born in January–March—attenuating at ages 11–17 years (p for interaction 0.06 and 0.04 between age period and birth month group) (table 2). Preterm born boys had an increase in odds of 25% for ADHD in both age periods, and no apparent relative age effects for other neuropsychiatric disorders. For the term population, relative age effects were substantial for ADHD, and stronger for girls than for boys both at ages 4–10 years (OR 1.78 (95% CI 1.63 to 1.95) vs 1.45 (95% CI 1.37 to 1.53, p for interaction between sex and birth month group <0001) and at ages 11–17 and also present for other neuropsychiatric disorders.

### Emotional disorders

As for the emotional disorders (anxiety/depression and adjustment/deprivation disorders), the patterns of prevalence over time differ substantially from neuropsychiatric disorders. Figure 3 displays prevalences by age and shows an extensive increase in the overall prevalence of these disorders in both term and preterm girls in adolescence and indicates relative age effects (higher prevalences in children born late in the year) in preterm girls in particular. Relative age effects (table 2) for preterm girls were most prominent at ages 4–10 years with ORs for adjustment disorders 1.76 (95% CI 1.14 to 2.72) and for anxiety and depression 1.40 (95% CI 1.04 to 1.89). For preterm boys and term boys and girls, relative age effects were negligible.

### Additional analyses

Results for the additional diagnostic outcomes are shown in online supplemental figure S2 and S3 and online supplemental table S4. For many of these, the prevalences were low, leading to relative effect estimates with broad CIs.

Results from analyses with three GA groups (online supplemental table S5) showed relative age effects in the moderate-late preterm group as well as for the very preterm participants, although in general stronger for the latter. Particularly strong relative age effects were seen among very preterm girls for ADHD at ages 11–17 (OR 3.42 (95% CI 2.00 to 5.85), and for adjustment/deprivation disorders at ages 4–10 (OR 4.43 (95% CI 1.47 to 13.3)).

Estimates did not change substantially after adjustment for a wider range of background variables (online supplemental table S6), nor after excluding individuals with CP, intellectual disability, from multiple births or with congenital birth defects (online supplemental table S7).

## Discussion

Children born late in the year had a higher risk of healthcare use with psychiatric diagnosis compared with those born early in the year, and such relative age effects were seen both for preterm and term-born boys and girls. Overall, the healthcare use with psychiatric diagnoses was higher in preterm born compared with their term counterparts, and the highest prevalences were observed for preterm born late in the year. Patterns of relative age effects varied for different categories of psychiatric diagnoses, with distinctive differences between neuropsychiatric and emotional disorders.

For neuropsychiatric diagnoses, relative age effects were prominent at ages 4–10 years. All over, preterm children and boys were more often registered with these conditions than their respective peers, but relative age effects were stronger for girls than boys in several categories. Our findings show an increased odds of 30%–80% for a diagnosis of ADHD at ages 4–10 years for the relatively youngest, and somewhat less for receiving a diagnosis of other neuropsychiatric disorders. The literature supports findings of relative age effects for ADHD^3^,^5^ and in areas related to cognitive, motor and socioemotional development.^1^ Also, for autism, there is some evidence of relative age effects.^32^ A possibility is that this could be driven by comorbidity with ADHD.^33^

Children born preterm are inherently susceptible to neuropsychiatric conditions.^34^ For school performance, studies have suggested that the relatively youngest preterm students fall behind.^23^,^25^ In our previous work, we showed persisting relative age effects for ADHD through adolescence for preterm born late in the year.^26^ In the present study, we did not see this distinct contrast in development over age between the preterm and term group; however, relative age effects were still present among participants aged 11–17 years. Prescriptions are likely a better measure than specialist care use of lasting relative age effects, in particular for ADHD, where prescription status and diagnosis correlate.^6^

Stronger relative age effects for girls than boys concerning ADHD have been demonstrated in several earlier studies.^3 6 35 36^ Little research has dealt with sex differences in preterm children, but findings from our previous study suggested larger relative effects for prescription of psychostimulants across adolescence for preterm girls,^26^ consistent with the tendency of the present findings. Larger relative age effects for neuropsychiatric conditions in the female part of the population may be due to a tendency to underdiagnose these kinds of conditions in girls,^37^ or the contrary, that boys are overdiagnosed with ADHD and related disorders.^38^ A possible explanation for findings of less relative age effects for preterm boys might be that they have a high baseline level of several of the studied conditions, and that the relative age markup is therefore not that clear. An alternative explanation could be that symptoms, possibly resulting in relative age effects, are explained by their prematurity, not resulting in a diagnosis.

For emotional disorders, girls overall were increasingly registered with such diagnoses through their teenage years, in accordance with previous research.^39^ The relatively youngest preterm girls were most at risk, with surprisingly strong relative age effects for adjustment and deprivation disorders through ages 4–17, and the same tendency for anxiety and depression in ages 4–10 years. We have not been able to find other studies assessing this. However, correlates can be drawn to our recent finding of relative age effects for antidepressant use among preterm girls in ages 10–14 years.^26^

In addition to the fact that adolescent girls seem to be particularly susceptible to emotional stress, both a younger relative age and prematurity are linked to an increased risk of being bullied.^40 41^ Furthermore, it appears that preterm children lack resilience to manage psychosocial stressors, which can result in a higher likelihood of experiencing adverse mental health outcomes.^42^ This may provide possible explanations for the observed risk in preterm girls relatively younger than their peers. However, for relative age effects in term born, other studies suggest similar results for emotional disorders across sex: a large Asian study found relative age effects for anxiety and depression, with decreasing effects after primary school,^9^ and a British study found relative age effects for depression in ages 4–15 years.^7^

### Strengths and limitations

Our registry data consist of a large natural population, containing all Norwegian citizens born from 1991 to 2012, with the possibility of long-term follow-up over several years.

We did not study comorbidity in particular. But as psychiatric comorbidity is common, one contact with healthcare could generate more than one diagnosis. Also, our data do not distinguish between diagnoses given in psychiatric and somatic healthcare, or between main and secondary diagnoses. Importantly, for the outcome of any F diagnosis, there is only one contribution. Not all individuals have contributed with the same number of years, but all contributed years are complete, ensuring that the data structure does not produce any biases for the relative age effects.

In conclusion, this study demonstrates that being more immature physically, cognitively and emotionally than one’s classmates may influence the youngest children in class in ways that lead to more contact with and diagnoses in psychiatric healthcare.

Our findings suggest that the burden following preterm birth is increased by young relative age, and that preterm born females are particularly vulnerable. In order to provide preterm children with the best possible follow-up, it is crucial to recognise their relative maturity. Relative age must be viewed as a modifiable risk factor that has the potential to contribute to more or less favourable mental health outcomes, and its effect can be altered by delaying school entry. This may be beneficial for children who mature later and are also the youngest in the class.

## supplementary material

10.1136/bmjpo-2024-003186 online supplemental file 1

## Data Availability

Data may be obtained from a third party and are not publicly available.
