# Supplementary material for: Relative age as a risk factor for psychiatric diagnoses in children born preterm and to term: a cohort study
Source: BMJ Paediatr Open. 2025 Apr 5;9(1):e003186. doi: 10.1136/bmjpo-2024-003186 (PMC11973766; doi:10.1136/bmjpo-2024-003186)
Supplement: online supplemental file 1 [file bmjpo-9-1-s001.pdf]

Content supplement

Table S1: The time frame of data availability. Page 2.

Table S2: Diagnostic groups, defined according to ICD-10. Page 3-4.

Figure S1: Directed acyclic graph (DAG), the relationship between relative age and mental health, showing possible confounding factors (adjusted for in the models). Page 5.

Table S3: Sociodemographic characteristics and perinatal variables of the subgroups: preterm with birth months January-March and October-December and term with birth months January-March and October-December. Page 6.

Figure S2 (supplement to figure 2): 1-year prevalences (in percent) of psychiatric Z diagnosis, and any F or Z diagnosis, from 4 to 17 years according to birth month group (only showing the first and fourth group), estimated by GEE analyses stratified by gestational age and sex, and with an interaction term between age (2-year intervals) and birth month group. Girls with green and black graphs to the left, boys in blue and black graphs to the right. Page 7.

Figure S3 (supplement to figure 2): 1-year prevalences (in percent) of additional diagnostic outcomes, from 4 to 17 years according to birth month group (only showing the first and fourth group), estimated by GEE analyses stratified by gestational age and sex, and with an interaction term between age (2-year intervals) and birth month group. Girls with green and black graphs to the left, boys in blue and black graphs to the right. Page 8.

Table S4 (supplement to table 2): Relative age effects - additional outcomes. Adjusted Odds Ratios (OR, with 95% CI) of annual psychiatric diagnoses at ages 4-10 and 11-17 years among the relatively younger preterm and term boys and girls born in October-December, compared to the relatively older groups born in January-March. Page 9.

Table S5: Relative age effects - gestational age subgroups. Adjusted Odds Ratios (OR, with 95% CI) of annual psychiatric diagnoses at ages 4-10 and 11-17 years among the relatively younger preterm and term boys and girls born in October-December, compared to the relatively older groups born in January-March, in three gestational age groups. Page 10.

Table S6. Relative age effects - sensitivity analysis with extended adjustment. Odds Ratios (OR, with 95% CI) of annual psychiatric diagnoses at ages 4-10 and 11-17 years among the relatively younger preterm and term boys and girls born in October-December, compared to the relatively older groups born in January-March, from three regression models; 1) unadjusted, 2) with main adjustment (participants' year of birth, sex, maternal age, parity and education) and 3) with extended adjustment (main model plus maternal relationship status and country of birth and paternal education). Page 11.

Table S7. Relative age effects - sensitivity analysis subpopulations. Odds Ratios (OR, with 95% CI) of annual psychiatric diagnosis (primary outcome) at ages 4-10 and 11-17 years among the relatively younger preterm and term boys and girls born in October-December, compared to the relatively older groups born in January-March, reported separately for. populations without individuals with CP, intellectual disability, from multiple births page and congenital birth defects. Page 12.

Table S8. Supplement to descriptives by birth month groups, table 2. Diagnostic outcomes for the entire period, regardless of number of observations. Page 13.

Table S1: Illustration of the time frame of data availability. Observable years for different cohorts are shown in red color. The National Patient registry started in 2008, and the data linkage used in the present study had updated data through 2017. Only full years of observation were included, so that the last year of observation started in the year in which the participants turned 17

The time frame of data availability made it difficult to ascertain first time events (For individuals who contributed with data from 2008, we could not know for sure whether they had been in contact with healthcare previously. This was particularly relevant for older individuals (e.g those born in 1994 who were 14 years old in 2008).

	Age during follow-up														
Cohort (birthyear)	4 yr	5 yr	6 yr	7 yr	8 yr	9 yr	10 yr	11 yr	12 yr	13 yr	14 yr	15 yr	16 yr	17 yr	No of obs in follow-up
1991	1995	1996	1997	1998	1999	2000	2001	2002	2003	2004	2005	2006	2007	2008	1
1992	1996	1997	1998	1999	2000	2001	2002	2003	2004	2005	2006	2007	2008	2009	2
1993	1997	1998	1999	2000	2001	2002	2003	2004	2005	2006	2007	2008	2009	2010	3
1994	1998	1999	2000	2001	2002	2003	2004	2005	2006	2007	2008	2009	2010	2011	4
1995	1999	2000	2001	2002	2003	2004	2005	2006	2007	2008	2009	2010	2011	2012	5
1996	2000	2001	2002	2003	2004	2005	2006	2007	2008	2009	2010	2011	2012	2013	6
1997	2001	2002	2003	2004	2005	2006	2007	2008	2009	2010	2011	2012	2013	2014	7
1998	2002	2003	2004	2005	2006	2007	2008	2009	2010	2011	2012	2013	2014	2015	8
1999	2003	2004	2005	2006	2007	2008	2009	2010	2011	2012	2013	2014	2015	2016	9
2000	2004	2005	2006	2007	2008	2009	2010	2011	2012	2013	2014	2015	2016	2017	9
2001	2005	2006	2007	2008	2009	2010	2011	2012	2013	2014	2015	2016	2017	2018	9
2002	2006	2007	2008	2009	2010	2011	2012	2013	2014	2015	2016	2017	2018	2019	9
2003	2007	2008	2009	2010	2011	2012	2013	2014	2015	2016	2017	2018	2019	2020	9
2004	2008	2009	2010	2011	2012	2013	2014	2015	2016	2017	2018	2019	2020	2021	9
2005	2009	2010	2011	2012	2013	2014	2015	2016	2017	2018	2019	2020	2021	2022	8
2006	2010	2011	2012	2013	2014	2015	2016	2017	2018	2019	2020	2021	2022	2023	7
2007	2011	2012	2013	2014	2015	2016	2017	2018	2019	2020	2021	2022	2023	2024	6
2008	2012	2013	2014	2015	2016	2017	2018	2019	2020	2021	2022	2023	2024	2025	5
2009	2013	2014	2015	2016	2017	2018	2019	2020	2021	2022	2023	2024	2025	2026	4
2010	2014	2015	2016	2017	2018	2019	2020	2021	2022	2023	2024	2025	2026	2027	3
2011	2015	2016	2017	2018	2019	2020	2021	2022	2023	2024	2025	2026	2027	2028	2
2012	2016	2017	2018	2019	2020	2021	2022	2023	2024	2025	2026	2027	2028	2029	1

Table S2: Diagnostic groups, defined according to ICD-10.

Outcomes	ICD-10 diagnostic codes
F diagnosis	Any diagnosis in the F (V) chapter
**Neuropsychiatric disorders:**	
ADHD	F90 Hyperkinetic disorders<br>• F90.0 Disturbance of activity and attention, F90.1 Hyperkinetic conduct disorder, F90.8 Other hyperkinetic disorders, F90.9 Hyperkinetic disorder, unspecified
Autism	F84 Pervasive developmental disorders<br>• F84.0 Childhood autism, F84.1 Atypical autism, F84.2 Rett syndrome, F84.3 Other childhood disintegrative disorder, F84.4 Overactive disorder associated with mental retardation and stereotyped movements, F84.5 Asperger syndrome, F84.8 Other pervasive developmental disorders, F84.9 Pervasive developmental disorder, unspecified
Other neuropsychiatric disorders	F80 Specific developmental disorders of speech and language<br>• F80.0 Specific speech articulation disorder, F80.1 Expressive language disorder, F80.2 Receptive language disorder, F80.3 Acquired aphasia with epilepsy, F80.8 Other developmental disorders of speech and language, F80.9 Developmental disorder of speech and language, unspecified<br>F81 Specific developmental disorders of scholastic skills<br>• F81.0 Specific reading disorder, F81.1 Specific spelling disorder, F81.2 Specific disorder of arithmetical skills, F81.3 Mixed disorder of scholastic skills, F81.8 Other developmental disorders of scholastic skills, F81.9 Developmental disorder of scholastic skills, unspecified<br>F82 Specific developmental disorders of motor function<br>F83 Mixed specific developmental disorders<br>F88 Other disorders of psychological development<br>F89 Unspecified disorder of psychological development<br>F95 Tic disorders<br>• F95.0 Trasient tic disorder, F95.1 Chronic motor or vocal tic disorder, F95.2 Combined vocal and multiple motor tic disorder (Tourette), F95.8 Other tic disorders, F95.9 Tic disorder, unspecified
**Emotional disorders:**	
Anxiety/ depression	F40 Phobic anxiety disorders<br>• F40.0 Agoraphobia, F40.1 Social phobias, F40.2 Specific (isolated) phobias, F40.8 Other phobic anxiety disorders, F40.9 Phobic anxiety disorder, unspecified)<br>F41 Other anxiety disorders<br>• F41.0 Panic disorder [episodic paroxysmal anxiety], F41.1 Generalized anxiety disorder, F41.2 Mixed anxiety and depressive disorder, F41.3 Other mixed anxiety disorders, F41.8 Other specified anxiety disorders, F41.9 Anxiety disorder, unspecified<br>F42 Obsessive-compulsive disorder<br>• F42.0 Predominantly obsessional thoughts or ruminations, F42.1 Predominantly compulsive acts, F42.2 Mixed obsessional thoughts and acts, F42.8 Other obsessive-compulsive disorders, F42.9 Obsessive-compulsive disorder, unspecified<br>F93 Emotional disorders with onset specific to childhood<br>• F93.0 Separation anxiety disorder of childhood, F93.1 Phobic anxiety disorder of childhood, F93.2 Social anxiety disorder of childhood, F93.3 Sibling rivalry disorder, F93.8 Other childhood emotional disorders, F93.9 Childhood emotional disorder, unspecified<br>F30 Manic episode<br>• F30.0 Hypomania, F30.1 Mania without psychotic symptoms, F30.2 Mania with psychotic symptoms, F30.8 Other manic episodes, F30.9 Manic episode, unspecified<br>F31 Bipolar affective disorder<br>• F31.0 Bipolar affective disorder, current episode hypomanic, F31.1 Bipolar affective disorder, current episode manic without psychotic symptoms, F31.2 Bipolar affective disorder, current episode manic with psychotic symptoms, F31.3 Bipolar affective disorder, current episode mild or moderate depression, F31.4 Bipolar affective disorder, current episode severe depression without psychotic symptoms, F31.5 Bipolar affective disorder, current episode severe depression with psychotic symptoms, F31.6 Bipolar affective disorder, current episode mixed, F31.7 Bipolar affective disorder, currently in remission, F31.8 Other bipolar affective disorders, F31.9 Bipolar affective disorder, unspecified<br>F32 Depressive episode<br>• F32.0 Mild depressive episode, F32.1 Moderate depressive episode, F32.2 Severe depressive episode without psychotic symptoms, F32.3 Severe depressive episode with psychotic symptoms, F32.8 Other depressive episodes, F32.9 Depressive episode, unspecified<br>F33 Recurrent depressive disorder<br>• F33.0 Recurrent depressive disorder, current episode mild, F33.1 Recurrent depressive disorder, current episode moderate, F33.2 Recurrent depressive disorder, current episode severe without psychotic symptoms, F33.3 Recurrent depressive disorder, current episode severe with psychotic symptoms, F33.4 Recurrent depressive disorder, currently in remission, F33.8 Other recurrent depressive disorders, F33.9 Recurrent depressive disorder, unspecified

	F34 Persistent mood disorders<br>• F34.0 Cyclothymia, F34.1 Dysthymia, F34.8 Other persistent mood disorders, F34.9 Persistent mood disorder, unspecified<br>F38 Other mood disorders<br>• F38.0 Other single mood disorders, F38.1 Other recurrent mood disorders, F38.8 Other specified mood disorders<br>F39 Unspecified mood disorders<br>F45 Somatoform disorders<br>• F45.0 Somatization disorder, F45.1 Undifferentiated somatoform disorder, F45.2 Hypochondriacal disorder, F45.3 Somatoform autonomic dysfunction, F45.4 Persistent somatoform pain disorder, F45.8 Other somatoform disorders, F45.9 Somatoform disorder, unspecified<br>F48 Other neurotic disorders<br>• F48.0 Neurasthenia, F48.1 Depersonalization-derealization syndrome, F48.8 Other specified neurotic disorders, F48.9 Neurotic disorder, unspecified
Adjustment/ deprivation disorders	F43 Reaction to severe stress, and adjustment disorders<br>• F43.0 Acute stress reaction, F43.1 Post-traumatic stress disorder, F43.2 Adjustment disorders, F43.8 Other reactions to severe stress, F43.9 Reaction to severe stress, unspecified<br>F44 Dissociative disorders<br>• F44.0 Dissociative amnesia, F44.1 Dissociative fugue, F44.2 Dissociative stupor, F44.3 Trance and possession disorders, F44.4 Dissociative motor disorders, F44.5 Dissociative convulsions, F44.6 Dissociative anaesthesia and sensory loss, F44.7 Mixed dissociative disorders, F44.8 Other dissociative disorders, F44.9 Dissociative disorder, unspecified
**Other diagnostic outcomes:**	
Behavioral disorders	F91 Conduct disorders<br>• F91.0 Conduct disorder confined to the family context, F91.1 Unsocialized conduct disorder, F91.2 Socialized conduct disorder, F91.3 Oppositional defiant disorder, F91.8 Other conduct disorders, F91.9 Conduct disorder, unspecified<br>F92 Mixed disorders of conduct and emotions<br>• F92.0 Depressive conduct disorder, F92.8 Other mixed disorders of conduct and emotions, F92.9 Mixed disorder of conduct and emotions, unspecified<br>F98 Other behavioural and emotional disorders with onset usually occurring in childhood and adolescence<br>• F98.0 Nonorganic enuresis, F98.1 Nonorganic encopresis, F98.2 Feeding disorder of infancy and childhood, F98.3 Pica of infancy and childhood, F98.4 Stereotyped movement disorders, F98.5 Stuttering, F98.6 Cluttering, F98.8 Other specified behavioural and emotional disorders with onset usually occurring in childhood and adolescence, F98.9 Unspecified behavioural and emotional disorders with onset usually occurring in childhood and adolescence
Relationship disorders	F94 Disorders of social functioning with onset specific to childhood and adolescence<br>• F94.0 Elective mutism, F94.1 Reactive attachment disorder of childhood, F94.2 Disinhibited attachment disorder of childhood, F94.8 Other childhood disorders of social functioning, F94.9 Childhood disorder of social functioning, unspecified
Eating disorders	F50 Eating disorders<br>• F50.0 Anorexia nervosa, F50.1 Atypical anorexia nervosa, F50.2 Bulimia nervosa, F50.3 Atypical bulimia nervosa, F50.4 Overeating associated with other psychological disturbances, F50.5 Vomiting associated with other psychological disturbances, F50.8 Other eating disorders, F50.9 Eating disorder, unspecified
Sleep disorders	F51 Nonorganic sleep disorders<br>• F51.0 Nonorganic insomnia, F51.1 Nonorganic hypersomnia, F51.2 Nonorganic disorder of the sleep-wake schedule, F51.3 Sleepwalking, F51.4 Sleep terrors, F51.5 Nightmares, F51.8 Other nonorganic sleep disorders, F51.9 Nonorganic sleep disorder, unspecified
Psychotic disorders	F20 Schizophrenia<br>• F20.0 Paranoid schizophrenia, F20.1 Disorganized schizophrenia, F20.2 Catatonic schizophrenia, F20.3 Undifferentiated schizophrenia, F20.5 Residual schizophrenia, F20.8 Other schizophrenia, F20.9 Unspecified schizophrenia.<br>F21 Schizotypal disorders<br>F22 Delusional disorders<br>F23 Brief psychotic disorder<br>F24 Shared psychotic disorder<br>F25 Schizoaffective disorders<br>• F25.0 Schizoaffective disorders, bipolare type, F25.1 Schizoaffective disorders, depressive type, F25.8 Other schizoaffective disorders, F25.0 Unspecified schizoaffective disorders.<br>F28 Other psychotic disorders<br>F29 Unspecified psychosis
Z diagnosis	Z00.4 General psychiatric examination, not elsewhere classified<br>Z03.2 Observation for suspected mental and behavioural disorders
F or Z diagnosis	Any F-diagnosis<br>Z00.4<br>Z03.2

Figure S1. Directed acyclic graph (DAG), the relationship between relative age and mental health, showing possible confounding factors (adjusted for in the models).

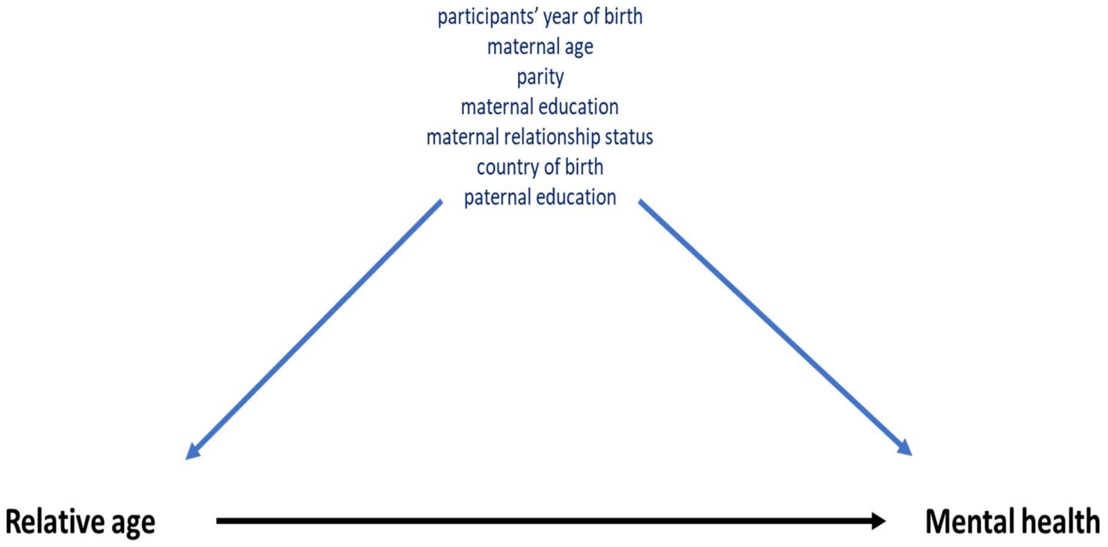

Table S3: Sociodemographic characteristics, perinatal variables and outcomes of the subgroups with birth months January - March and October – December:

	Term[a], born in Jan-March[b]	Term[a], born in Oct-Dec[c]	Preterm[d], born in Jan-March[c]	Preterm[d], born in Oct-Dec[d]	All groups
	n (%) / Mean (SD)	n (%) / Mean (SD)	n (%) / Mean (SD)	n (%) / Mean (SD)	n (%) / Mean (SD)
**Total:**	255 884 (48,2)	236 802 (44,6)	19 147 (3,6)	18 594 (3,5)	530 427 (100)
**Gender:**					
Boys	129 863 (50,8)	119 942 (50,7)	10 133 (52,9)	9 887 (53,2)	269 825 (50,9)
Girls	126 021 (49,3)	116 860 (49,4)	9 014 (47,1)	8 707 (46,8)	260 602 (49,1)
**Mean birthweight, g (SD):**	3 571 (494)	3 573 (495)	2 395 (673)	2 373 (690)	3 488 (595)
**Gestational age:**					
Mean weeks (SD)	40,0 (1,2)	40,0 (1,2)	34,6 (2,4)	34,5 (2,6)	39,6 (1,9)
**Small for gestational age[e]:**	8 977 (3,5)	8 235 (3,5)	2 970 (15,5)	2 959 (15,9)	23 141 (4,4)
**Large for gestational age[f]:**	12 283 (4,8)	11 224 (4,7)	1 160 (6,1)	1 112 (6,0)	25 779 (4,9)
**Mother's relationship status:**					
Married/ cohabitant	235 292 (92,0)	218 056 (92,1)	17 256 (90,1)	16 874 (90,8)	487 478 (91,9)
Other	20 592 (8,1)	18 746 (7,9)	1 891 (9,9)	1 720 (9,3)	42 949 (8,1)
**Multiple births:**					
Singeltons	250 276 (97,8)	231 900 (97,9)	14 663 (76,6)	14 151 (76,1)	510 990 (96,3)
Multiples	5 608 (2,2)	4 902 (2,1)	4 484 (23,4)	4 443 (23,9)	19 437 (3,7)
**Parity:**					
Primiparae	101 529 (39,7)	98 835 (41,7)	9 171 (47,9)	9 491 (51,0)	219 026 (41,3)
Para 1	95 184 (37,2)	83 472 (35,3)	5 800 (30,3)	5 233 (28,1)	189 689 (35,8)
Para 2	42 646 (16,7)	38 657 (16,3)	2 727 (14,2)	2 522 (13,6)	86 552 (16,3)
Para 3	11 678 (4,6)	11 007 (4,7)	946 (4,9)	885 (4,8)	24 516 (4,6)
Para 4 or more	4 847 (1,9)	4 831 (2,0)	503 (2,6)	463 (2,5)	10 644 (2,0)
**Maternal mean age, years (SD):**	29,1 (5,1)	29,2 (5,2)	29,4 (5,3)	29,5 (5,5)	29,2 (5,2)
**Maternal education:**					
Lower secondary education	58 466 (22,9)	55 218 (23,3)	5 087 (26,6)	4 858 (26,1)	123 629 (23,3)
Upper secondary education	94 682 (37,0)	85 800 (36,2)	7 118 (37,2)	7 018 (37,7)	194 618 (36,7)
Higher education	102 736 (40,2)	95 784 (40,5)	6 942 (36,3)	6 718 (36,1)	212 180 (40,0)
**Maternal country of birth:**					
Norway	211 665 (82,7)	192 307 (81,2)	15 743 (82,2)	15 120 (81,3)	434 835 (82,0)
Other	35 338 (13,8)	36 979 (15,6)	2 684 (14,0)	2 786 (15,0)	77 787 (14,7)
**Congenital birth defects:**	9 628 (3,8)	8 636 (3,7)	1 531 (8,0)	1 494 (8,0)	21 289 (4,0)

[a] Gestational age 37 weeks and 0 days to 41 weeks and 6 days
[b] Born in January to March
[c] Born in October to December
[d] Gestational age 23 weeks and 0 days to 36 weeks and 6 days
[e] Birth weight < 2,5th percentile for gestational age
[f] Birth weight > 97,5th percentile for gestational age

Figure S2: 1-year prevalences (in percent) of psychiatric Z diagnosis, and any F or Z diagnosis, from 4 to 17 years according to birth month group (only showing the first and fourth group), estimated by GEE analyses stratified by gestational age and with an interaction term between age (2-year intervals) and birth month group. Girls with green and black graphs to the left, boys in blue and black graphs to the right.

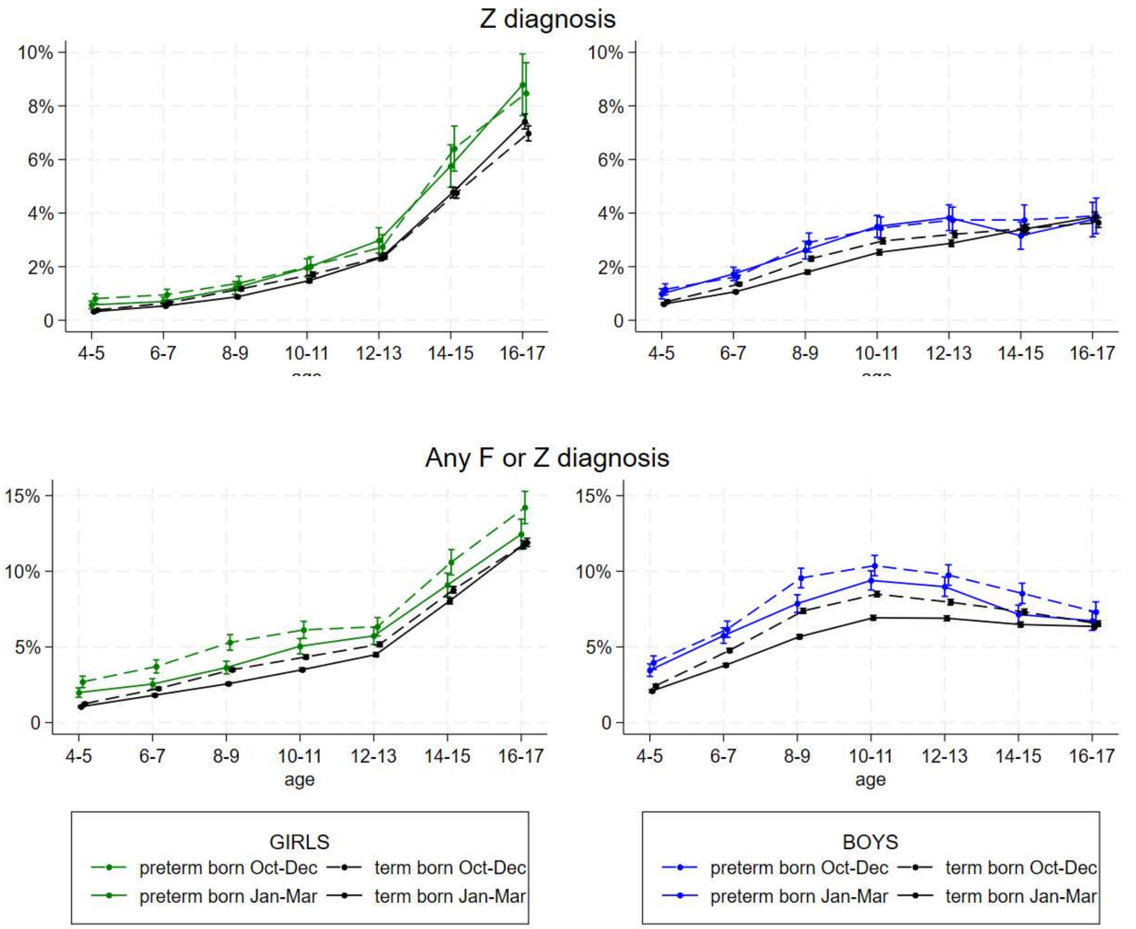

Figure S3 (supplement to figure 2): 1-year prevalences (in percent) of additional diagnostic outcomes, from 4 to 17 years according to birth month group (only showing the first and fourth group), estimated by GEE analyses stratified by gestational age and sex, and with an interaction term between age (2-year intervals) and birth month group. Girls with green and black graphs to the left, boys in blue and black graphs to the right.

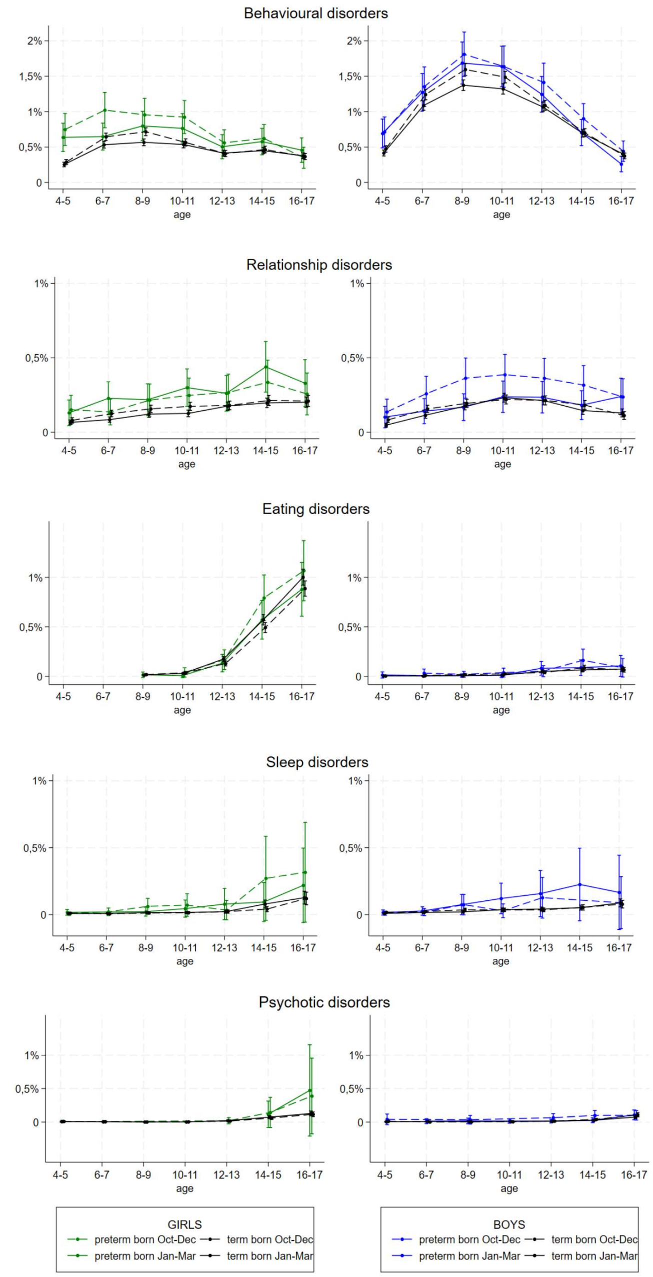

Table S4 (supplement to table 2): Relative age effects - additional outcomes. Adjusted Odds Ratios (OR, with 95% CI) of annual psychiatric diagnoses at ages 4-10 and 11-17 years among the relatively younger preterm and term boys and girls born in October-December, compared to the relatively older groups born in January-March.

	Preterm						Term					
	Boys			Girls			Boys			Girls		
	**OR**	95% CI		**OR**	95% CI		**OR**	95% CI		**OR**	95% CI	
**Behavioral disorders**												
4-10 y:	**0,99**	0,81	1,20	**1,27**	0,98	1,65	**1,13**	1,06	1,20	**1,20**	1,10	1,30
11-17 y:	**1,34**	1,06	1,68	**1,05**	0,77	1,43	**1,03**	0,96	1,11	**1,00**	0,91	1,10
**Relationship disorders**												
4-10 y:	**1,48**	0,89	2,47	**0,81**	0,47	1,39	**1,20**	1,01	1,42	**1,32**	1,09	1,61
11-17 y:	**1,69**	1,07	2,68	**0,90**	0,57	1,42	**1,09**	0,93	1,29	**1,10**	0,93	1,30
**Eating disorders**												
4-10 y:	**0,70**	0,26	1,84	**2,04**	0,53	7,85	**1,64**	1,14	2,35	**0,83**	0,53	1,30
11-17 y:	**0,46**	0,15	1,37	**1,39**	0,57	3,41	**0,91**	0,68	1,24	**0,88**	0,64	1,21
**Sleep disorders**												
4-10 y:	**3,11**	0,40	23,88	**0,42**	0,04	4,29	**1,78**	1,00	3,16	**1,11**	0,67	1,85
11-17 y:	**1,18**	0,58	2,42	**1,36**	1,00	1,83	**1,09**	0,83	1,42	**0,88**	0,80	0,97
**Psychotic disorders**												
4-10 y:	**1,37**	0,22	8,38	**1,02**	0,47	2,24	**0,72**	0,37	1,42	**1,22**	0,57	2,58
11-17 y:	**1,78**	0,86	3,68	**1,02**	0,47	2,24	**1,36**	1,03	1,81	**0,86**	0,68	1,08
**Z diagnosis**												
4-10 y:	**1,07**	0,93	1,23	**1,29**	1,07	1,56	**1,24**	1,19	1,30	**1,28**	1,20	1,36
11-17 y:	**1,04**	0,91	1,19	**1,02**	0,89	1,16	**1,06**	1,02	1,11	**1,01**	0,97	1,05
**Any (F or Z) diagnosis**												
4-10 y:	**1,14**	1,04	1,25	**1,43**	1,27	1,61	**1,26**	1,22	1,30	**1,28**	1,23	1,33
11-17 y:	**1,15**	1,05	1,25	**1,16**	1,06	1,27	**1,13**	1,10	1,16	**1,10**	1,07	1,13

Table S5: Relative age effects - gestational age subgroups. Adjusted Odds Ratios (OR, with 95% CI) of annual psychiatric diagnoses at ages 4-10 and 11-17 years among the relatively younger preterm and term boys and girls born in October-December, compared to the relatively older groups born in January-March, in three gestational age groups.

Boys

	**F diagnosis**			**ADHD**			**Autism**			**Other neuropsych dis**			**Anxiety/ depression**			**Adjust/ depriv. dis**		
**GA 23+0-31+6**	**OR**	95% CI		**OR**	95% CI		**OR**	95% CI		**OR**	95% CI		**OR**	95% CI		**OR**	95% CI	
4-10 y:	**1,23**	0,98	1,55	**1,35**	0,96	1,91	**0,99**	0,53	1,84	**1,47**	1,00	2,17	**1,53**	0,79	2,94	**0,85**	0,32	2,26
11-17 y:	**1,25**	0,99	1,57	**1,03**	0,74	1,45	**1,42**	0,74	2,74	**1,18**	0,81	1,72	**1,44**	0,93	2,23	**1,14**	0,53	2,44
**GA 32+0-36+6**																		
4-10 y:	**1,12**	1,00	1,25	**1,25**	1,05	1,49	**1,21**	0,89	1,66	**0,95**	0,78	1,16	**0,97**	0,72	1,31	**0,76**	0,50	1,16
11-17 y:	**1,13**	1,02	1,26	**1,29**	1,10	1,51	**1,37**	1,04	1,80	**1,04**	0,87	1,25	**1,01**	0,82	1,24	**0,87**	0,63	1,19
**GA 37+0-41+6**																		
4-10 y:	**1,26**	1,22	1,30	**1,45**	1,37	1,53	**1,11**	1,01	1,21	**1,36**	1,28	1,45	**1,12**	1,02	1,22	**1,13**	1,00	1,27
11-17 y:	**1,17**	1,13	1,20	**1,34**	1,27	1,40	**1,08**	1,00	1,17	**1,20**	1,13	1,27	**1,07**	1,02	1,13	**1,09**	1,00	1,19

Girls

	**F diagnosis**			**ADHD**			**Autism**			**Other neuropsych dis**			**Anxiety/ depression**			**Adjust/ depriv. dis**		
**GA 23+0-31+6**	**OR**	95% CI		**OR**	95% CI		**OR**	95% CI		**OR**	95% CI		**OR**	95% CI				
4-10 y:	**1,38**	1,05	1,83	**1,49**	0,92	2,44	**1,79**	0,61	5,27	**1,82**	1,09	3,04	**1,17**	0,61	2,23	**4,43**	1,47	13,3
11-17 y:	**1,39**	1,09	1,77	**3,42**	2,00	5,85	**0,68**	0,29	1,61	**1,42**	0,86	2,33	**1,31**	0,89	1,92	**1,97**	1,17	3,33
**GA 32+0-36+6**																		
4-10 y:	**1,43**	1,23	1,66	**1,55**	1,16	2,07	**0,87**	0,43	1,77	**1,40**	1,03	1,92	**1,46**	1,05	2,04	**1,44**	0,90	2,31
11-17 y:	**1,11**	1,00	1,24	**0,93**	0,73	1,19	**0,96**	0,58	1,60	**0,83**	0,64	1,07	**1,15**	0,99	1,34	**1,27**	1,01	1,60
**GA 37+0-41+6**																		
4-10 y:	**1,27**	1,21	1,33	**1,78**	1,63	1,95	**1,11**	0,91	1,34	**1,27**	1,15	1,40	**1,09**	0,98	1,20	**1,06**	0,93	1,21
11-17 y:	**1,12**	1,09	1,16	**1,49**	1,39	1,59	**1,23**	1,07	1,41	**1,23**	1,14	1,33	**1,08**	1,03	1,12	**1,03**	0,96	1,09

Table S6. Relative age effects - sensitivity analysis with extended adjustment. Odds Ratios (OR, with 95% CI) of annual psychiatric diagnoses at ages 4-10 and 11-17 years among the relatively younger preterm and term boys and girls born in October-December, compared to the relatively older groups born in January-March, from three regression models; 1) unadjusted (OR_u), 2) with main adjustment (participants' year of birth, sex, maternal age, parity and education) (OR_a) and 3) with extended adjustment (main model plus maternal relationship status and country of birth and paternal education, for all individuals with complete data) (OR_{ses}).

	Preterm									Term								
	**OR_u**	95% CI		**OR_a**	95% CI		**OR_{ses}**	95% CI		**OR_u**	95% CI		**OR_a**	95% CI		**OR_{ses}**	95% CI	
**F diagnosis**																		
4-10 y:	**1,25**	1,15	1,36	**1,24**	1,15	1,35	**1,25**	1,16	1,36	**1,26**	1,23	1,30	**1,26**	1,23	1,30	**1,27**	1,23	1,30
11-17 y:	**1,17**	1,08	1,25	**1,17**	1,09	1,25	**1,18**	1,09	1,26	**1,14**	1,12	1,17	**1,15**	1,12	1,17	**1,15**	1,13	1,18
**ADHD**																		
4-10 y:	**1,35**	1,18	1,56	**1,36**	1,18	1,55	**1,37**	1,20	1,58	**1,52**	1,45	1,59	**1,52**	1,45	1,60	**1,54**	1,47	1,61
11-17 y:	**1,23**	1,08	1,40	**1,24**	1,09	1,41	**1,26**	1,11	1,43	**1,36**	1,31	1,42	**1,38**	1,32	1,43	**1,39**	1,34	1,44
**Autism**																		
4-10 y:	**1,21**	0,93	1,57	**1,19**	0,92	1,54	**1,19**	0,92	1,55	**1,12**	1,03	1,22	**1,11**	1,02	1,21	**1,11**	1,02	1,20
11-17 y:	**1,26**	1,00	1,59	**1,24**	0,99	1,57	**1,24**	0,99	1,57	**1,13**	1,05	1,21	**1,12**	1,04	1,20	**1,11**	1,04	1,20
**Other neuropsych disorders**																		
4-10 y:	**1,18**	1,01	1,37	**1,16**	1,00	1,35	**1,17**	1,00	1,36	**1,35**	1,28	1,43	**1,34**	1,27	1,42	**1,35**	1,28	1,43
11-17 y:	**1,04**	0,90	1,20	**1,05**	0,91	1,20	**1,06**	0,92	1,21	**1,20**	1,15	1,26	**1,21**	1,15	1,26	**1,21**	1,16	1,27
**Depression/ anxiety**																		
4-10 y:	**1,22**	1,00	1,49	**1,21**	0,99	1,49	**1,22**	0,99	1,49	**1,12**	1,05	1,19	**1,12**	1,05	1,20	**1,12**	1,05	1,20
11-17 y:	**1,15**	1,02	1,30	**1,15**	1,02	1,29	**1,15**	1,03	1,30	**1,08**	1,04	1,12	**1,08**	1,05	1,12	**1,09**	1,05	1,12
**Adjustment/ deprivation dis**																		
4-10 y:	**1,18**	0,88	1,58	**1,18**	0,88	1,59	**1,19**	0,88	1,60	**1,11**	1,01	1,21	**1,11**	1,01	1,22	**1,11**	1,01	1,22
11-17 y:	**1,14**	0,95	1,37	**1,15**	0,96	1,37	**1,15**	0,96	1,38	**1,05**	0,99	1,10	**1,05**	1,00	1,10	**1,05**	1,00	1,11

Table S7. Relative age effects - sensitivity analysis subpopulations. Odds Ratios (OR, with 95% CI) of annual psychiatric diagnosis (primary outcome) at ages 4-10 and 11-17 years among the relatively younger preterm and term boys and girls born in October-December, compared to the relatively older groups born in January-March, reported separately for. populations without individuals with CP, intellectual disability, from multiple births page and congenital birth defects. The three adjustment models are the same as in Table S6.

		F diagnosis preterm								
**Population:**		**OR_u**	95% CI		**OR_a**	95% CI		**OR_{ses}**	95% CI	
**Without CP**	4-10 y:	**1,25**	1,16	1,36	**1,24**	1,15	1,35	**1,25**	1,16	1,36
	11-17 y:	**1,17**	1,09	1,26	**1,17**	1,10	1,26	**1,19**	1,10	1,28
**Without intellectual disability**	4-10 y:	**1,26**	1,16	1,36	**1,25**	1,15	1,35	**1,26**	1,16	1,37
	11-17 y:	**1,17**	1,09	1,26	**1,17**	1,09	1,26	**1,19**	1,10	1,28
**Without multiple births**	4-10 y:	**1,20**	1,10	1,31	**1,19**	1,09	1,30	**1,19**	1,09	1,31
	11-17 y:	**1,15**	1,06	1,24	**1,15**	1,06	1,24	**1,15**	1,07	1,25
**Without congenital birth defects**	4-10 y:	**1,28**	1,17	1,39	**1,27**	1,17	1,38	**1,28**	1,17	1,40
	11-17 y:	**1,18**	1,09	1,27	**1,18**	1,10	1,26	**1,19**	1,11	1,29
		**F diagnosis term**								
**Population:**		**OR_u**	95% CI		**OR_a**	95% CI		**OR_{ses}**	95% CI	
**Without CP**	4-10 y:	**1,26**	1,23	1,29	**1,26**	1,23	1,29	**1,27**	1,24	1,30
	11-17 y:	**1,14**	1,12	1,16	**1,14**	1,12	1,17	**1,15**	1,13	1,18
**Without intellectual disability**	4-10 y:	**1,27**	1,23	1,3	**1,27**	1,23	1,3	**1,28**	1,24	1,31
	11-17 y:	**1,14**	1,12	1,16	**1,14**	1,12	1,17	**1,15**	1,13	1,18
**Without multiple births**	4-10 y:	**1,26**	1,22	1,29	**1,26**	1,22	1,29	**1,27**	1,23	1,30
	11-17 y:	**1,14**	1,12	1,17	**1,14**	1,12	1,17	**1,15**	1,13	1,18
**Without congenital birth defects**	4-10 y:	**1,26**	1,22	1,29	**1,26**	1,22	1,29	**1,26**	1,23	1,30
	11-17 y:	**1,14**	1,11	1,16	**1,14**	1,12	1,17	**1,15**	1,13	1,18

Table S8. Supplement to descriptives by birth month groups, table 1. Diagnostic outcomes for the entire period, regardless of number of observations.

	Jan-March[a]	Apr-June[b]	July-Sept[c]	Oct-Dec[d]	**All**
	n (%)	n (%)	n (%)	n (%)	**n (%)**
**Diagnostic groups**:					
F diagnosis	30 666 (11,2)	33 132 (11,5)	34 897 (12,2)	33 007 (13,0)	**131 702 (11,9)**
ADHD	7 592 (2,8)	8 633 (3,0)	9 928 (3,5)	10 061 (4,0)	**36 214 (3,3)**
Autism	2 466 (0,9)	2 668 (0,9)	2 822 (1,0)	2 726 (1,1)	**10 682 (1,0)**
Other neuropsych disorders	6 382 (2,3)	7 007 (2,4)	7 744 (2,7)	7 565 (3,0)	**28 698 (2,6)**
Anxiety/ depression	11 069 (4,0)	11 064 (4,1)	11 961 (4,2)	11 118 (4,4)	**46 012 (4,2)**
Adjustment/ depr disorders	4 951 (1,8)	5 169 (1,8)	5 365 (1,9)	4 840 (1,9)	**20 325 (1,8)**
Behavioural disorders	5 901 (2,2)	6 277 (2,2)	6 600 (2,3)	6 204 (2,4)	**24 982 (2,3)**
Relationship disorders	951 (0,4)	949 (0,3)	1 092 (0,4)	1 051 (0,4)	**4 043 (0,4)**
Eating disorders	1 327 (0,5)	1 325 (0,5)	1 313 (0,5)	1 158 (0,5)	**5 123 (0,5)**
Sleep disorders	314 (0,1)	302 (0,1)	353 (0,1)	321 (0,1)	**1 290 (0,1)**
Psychotic disorders	349 (0,1)	373 (0,1)	408 (0,1)	325 (0,1)	**1 455 (0,1)**
Intellectual disability	1 439 (0,5)	1 609 (0,6)	1 493 (0,5)	1 499 (0,6)	**6 040 (0,6)**
Cerebral palsy (CP)	895 (0,3)	876 (0,3)	965 (0,3)	910 (0,4)	**3 646 (0,3)**
Z diagnosis	17 643 (6,4)	18 414 (6,4)	19 178 (6,7)	17 621 (6,9)	**72 856 (6,6)**
F or Z diagnosis	35 799 (13,1)	38 227 (13,2)	39 995 (13,9)	37 544 (14,8)	**151 565 (13,7)**
