# Supplementary material for: Relative age as a risk factor for psychiatric diagnoses in children born preterm and to term: a cohort study
Source: BMJ Paediatr Open. 2025 Apr 5;9(1):e003186. doi: 10.1136/bmjpo-2024-003186 (PMC11973766; doi:10.1136/bmjpo-2024-003186)
Supplement: Supplementary file 2 [file bmjpo-2024-003186.reviewer_comments.pdf]

PEER REVIEW HISTORY

BMJ Paediatrics Open publishes all reviews undertaken for accepted manuscripts. Reviewers are asked to complete a checklist review form and are provided with free text boxes to elaborate on their assessment. These free text comments are reproduced below.

ARTICLE DETAILS

**TITLE (PROVISIONAL)**	Relative age as a risk factor for psychiatric diagnoses in children born preterm and to term: a cohort study
**AUTHORS**	Bachmann, Christine Strand; Risnes, Kari; Bjørngaard, Johan Håkon; Schei, Jorun; Nilsen, Sara Marie; Pape, Kristine

VERSION 1 - REVIEW

**REVIEWER NAME**	***ShankariR Arunanthy***
**REVIEWER AFFILIATION**	Royal Prince Alfred Hospital
**REVIEWER CONFLICT OF INTEREST**	N/A
**DATE REVIEW RETURNED**	04-Dec-2024

**GENERAL COMMENTS**	I think this is a really interesting and important topic!<br><br>Things to consider<br>1. Results section of abstract is vague. Please report key findings with key stats<br>2. What this study adds? - Again vague. Please add some clear and specific findings<br>3. Age divisions - why the division at 4-10 and 11-17? To me this is developmentally inappropriate and is possibly confounding your results. This is best reflected in Figure 2 where there are sharp changes in numbers of ICD diagnoses at particular timepoints - these timepoints are much more consistent with what we know about neurodevelopment and when different neurodevelopmental or psychiatric diagnoses might present and therefore be diagnosed. If you divided your results accordingly you might find clearer patterns<br>4. Primary outcome (P11; L16) - why results not shown?<br>5. It would be ideal for Figure 2 to be included in the main paper. It is perhaps the most convincing and interesting part of your paper.<br>6. Discussion (P14; L14-15) "many of which contain an aspect of maturity" - what does this mean?<br>7. Discussion (P14; L20-21) -there are a few studies on ASD and relative age effect. might be worth clarifying for your paper.<br>8. Discussion (P14; L23) - what evidence if any due to have for overlap between ASD and ADHD<br>9. Discussion (P14; L26-38) - is it possible that this is confounded by your age brackets and having different age brackets would show this up as preterm children more likely to be more severe and diagnosed earlier (could be confounded by clinicians deciding symptoms are as a result of prematurity rather than neuropsych dx; as you have suggested further on)<br>10. Discussion (P14; L42): dealt not delt

	11. Discussion (P15; L22): bullied not bullyed<br>12. Strengths and limitations: this is very limited. would be better to expand if possible.<br><br>Otherwise excellent and thought provoking!

**REVIEWER NAME**	***Linda Tran***
**REVIEWER AFFILIATION**	Rivendell Child and Adolescent Unit
**REVIEWER CONFLICT OF INTEREST**	N/A
**DATE REVIEW RETURNED**	24-Dec-2024

**GENERAL COMMENTS**	Your study utilizes a large population sample which provides important insights into long-term trajectories and outcomes based on the relative age paradigm. The amplification of the vulnerability for the relative younger children compared to their peers is both theoretically robust and practical e.g. regarding when to commence school or repeating a child at school etc. You take into consideration the effects of prematurity, gender and relative age in regard to "psychiatric issues". However, it is not a clear paper and the focus needs to be sharpened to bring out the points.<br><br>Specifics Suggestions:<br><br>Page 3, L 40: punctuation- full stop AFTER reference<br><br>P3, L56: more details on what is meant that there is a collaboration with families? Does this mean they had any input e.g in study design ?<br><br>Page 4, L 35, Exposures: how did you arrive at these subgroups? It seems somewhat arbitrary. Why didn't you eg. use late preterm as a subgroup which is 34+0-36+6?<br><br>Page 4, L 43: "In Norway, birth month is a good measure of relative age, since only about 0,6 % of Norwegian children delay starting school every year." – do you also mean that a school year in Norway starts in January of each year?<br><br>Table 2:<br>- typo in eationg disorder<br>- you refer to p values in the text but unsure where to find them in your data<br>- if I understand the table correctly, I arrive at following conclusions:<br>o term boys have higher OR than preterm boys for all disorders except for ASD<br>o preterm girls have higher OR than term girls for all disorders except for ADHD and for ASD<br>o term boys and girls have higher OR for ADHD than their preterm counterparts<br><br><br>Page 6, L 5-14: What is an interaction term? A clearer explanation would be helpful

	Page 7, L10: “Table S3 displays the subpopulation of 530427 individuals born in January-March and October-December.” – do these 2 groups differ from the April – September groups?<br><br>Page 12, L 53: “The prevalence of healthcare use with psychiatric diagnoses was higher in preterm born compared to their term counterparts”- term boys have higher OR than preterm boys for all disorders except for ASD<br><br>Page 13, L5: “Preterm children and boys, in particular, were more often registered with these (neuropsychiatric) conditions “– from table 2 it seems that term boys have higher OR than preterm boys for all disorders except for ASD<br><br>Page 13, L42, typo: delt-dealt<br><br>Page 13, L 45: “prescription of psychostimulants across adolescence for preterm girls”- is this trying to explain that the OR for ADHD is lower aged 11-17 compared to 4-10 in girls? But this reduction in OR is for both genders and for term and preterm. Are there alternate explanations e.g. less ADHD symptoms due to maturity or e.g. kids leaving school earlier etc.?<br>P13, L 51: “boys are overdiagnosed with ADHD”- how do you explain that in table 2 girls (term and preterm) have higher OR for ADHD than boys for age 4-17 ?<br>P14, L16: punctuation- full stop AFTER reference<br>P14, L22: typo “bullyed”- bullied<br>P14, L 22: “Furthermore, it appears that preterm children lack resilience to manage psychosocial stressors, which can result in a higher likelihood of experiencing adverse mental health outcomes.” – Table 2 seems to show the contrary; that term boys have higher OR than preterm boys for all disorders except for ASD and term children have higher OR for ADHD than their preterm counterparts<br>P 14, L 25: “This may provide possible explanations for the observed risk in preterm girls relatively young.” – unsure if this sentence is complete? Or do you mean something like ‘preterm girls who are relatively younger than their peers’?<br><br>This paper would be considerably enhanced by answering the following questions:<br>1. Would you advise, based on this data, that premature boys be started at school later in the year or the following year than term boys?<br>2. Would you advise, based on this data, that premature girls be started at school later in the year or the following year than term girls?<br>3. Are preterm girls more vulnerable to a lower and neurodevelopmentally complicated trajectory than boys?<br>4. Is the relationship between psychiatric disorder and neurodevelopmental disorder categorical or dimension ie two aspects of the same vulnerability or at extremes are they different in kind?<br>5. Does prematurity confer any advantage or relative advantage on any subgroup?

	This paper now needs minor revisions to sharpen the message. I am happy to review the revised version and hope the comments are helpful.

VERSION 1 – AUTHOR RESPONSE

Reviewer: 1
Dr. Shankari Arunanthy, Royal Prince Alfred Hospital
Comments to the Author
I think this is a really interesting and important topic!

Things to consider
1. Results section of abstract is vague. Please report key findings with key stats

This is changed in the manuscript due to the comment:

Old text:

***Results:** Of 1109411 individuals, 7% were born preterm and 23% born October-December. Relative age effects for any psychiatric diagnosis were seen for both preterm and term-born boys and girls, strongest in ages 4-10 years. The highest prevalences were seen in preterm born late in the year.*

New text:

***Results:** Of 1109411 individuals, 7% were born preterm. Relative age effects for psychiatric diagnosis and specific diagnose groups were seen for both preterm and term-born boys and girls with strongest estimates for any psychiatric diagnosis in the relatively younger preterm girls born in October-December, compared to the relatively older preterm girls born in January-March (OR 1,43 (95% CI, 1,25-1,63) at ages 4-10 years).*

2. What this study adds? - Again vague. Please add some clear and specific findings

This is changed in the manuscript due to the comment.

Old text:

***What this study adds:** Relative age effects were widely demonstrated for psychiatric diagnoses.*

***How this study might affect research, practice or policy:** The excess risk for those born late in the year added to already existing adversity in preterm children, emphasizing the need for additional consideration related to school and societal structures.*

New text:

***What this study adds:** For both term born and preterm born, relative age effects were widely demonstrated for healthcare use related to psychiatric diagnoses. The observed relative age effect in preterm adds to the already known mental health vulnerability in this group.*

***How this study might affect research, practice or policy:** Excess mental health burden in preterm children born late in the year emphasize the need for additional consideration related to school and societal structures in preterm born.*

3. Age divisions - why the division at 4-10 and 11-17? To me this is developmentally inappropriate and is possibly confounding your results. This is best reflected in Figure 2 where there are sharp changes in numbers of ICD diagnoses at particular timepoints - these timepoints are much more consistent with what we know about neurodevelopment and when different neurodevelopmental or psychiatric diagnoses might present and therefore be diagnosed. If you divided your results accordingly you might find clearer patterns

This is a valid point. We agree that age is a very important dimension regarding diagnosis (presence) of mental health issues - in general and more specifically for children born preterm. In addition, age is highly relevant for relative age effects, since we expect these to be more prominent at a lower age and in relation to starting school (in Norway school start is at 6 years and we wanted to show the transition from pre-school to school). This is why we have chosen to include age in two-year periods in all main analyses, and also to present all main results according to age (Figures 2, 3, S1 and S2). These figures may be used to evaluate the presence and impact of relative age among preterm and term born boys and girls at different ages. This paper includes many exposure groups and outcomes, and when quantifying relative age effects, we wanted to minimize the impact of random findings (due to multiple testing) and also to be able to describe and communicate the bigger picture to the reader. Also, one important aim was to evaluate whether relative age effects diminished (or increased) by age. The two-period categorization of age for the second analytical approach was chosen as a "best way" to do this.

In order to clarify the rationale of our choices, we made some changes to the text in the description of the second analytical approach in the statistics section:

In this analysis, follow-up age (at outcome assessment) was divided in only two periods (4-10 and 11-17 years), in order to provide robust findings but also to allow a statistical evaluation of changes in relative age effects over time (by including an interaction term between birth month group and the age periods).

4. Primary outcome (P11; L16) - why results not shown?

In figures 2 and 3, we chose not to show the results for the two groups in between the relatively oldest and the relatively youngest (this would involve four more lines in each figure) to simplify the results and illustrate the main findings clearer.

For visibility, only January-March and October-December are shown. This is specified in the statistics section.

5. It would be ideal for Figure 2 to be included in the main paper. It is perhaps the most convincing and interesting part of your paper.

Figure 2 is intended to be part of the paper, but the submission system did not allow it to be submitted as part of the paper itself. We are pointing out this to the editor for this resubmission, thank you for pointing to this.

6. Discussion (P14; L14-15) "many of which contain an aspect of maturity" - what does this mean?

We agree that this sentence is unclear - the wording is now changed in the text, see point 7.

7. Discussion (P14; L20-21) -there are a few studies on ASD and relative age effect. might be worth clarifying for your paper.

Thanks – this is now included in the text:

Old text:

The literature supports findings of relative age effects for ADHD (3-5). For other neuropsychiatric diagnoses, many of which contain an aspect of maturity, earlier studies have shown relative age effects in areas related to cognitive, motor and socio-emotional development (1).

We are not familiar with studies assessing relative age effects for a diagnosis of autism, which was suggested for preterm boys by the current study. A possibility is that effects of relative age on being diagnosed with autism could be driven by comorbidity with ADHD (32).

New text:

The literature supports findings of relative age effects for ADHD (3-5) and in areas related to cognitive, motor and socio-emotional development (1). Also, for autism there is some evidence of relative age effects (32). A possibility is that this could be driven by comorbidity with ADHD (33).

8. Discussion (P14; L23) - what evidence if any due to have for overlap between ASD and ADHD

If our understanding of the reference is correct, this paper points to comorbidity between these conditions.

9. Discussion (P14; L26-38) - is it possible that this is confounded by your age brackets and having different age brackets would show this up as preterm children more likely to be more severe and diagnosed earlier (could be confounded by clinicians deciding symptoms are as a result of prematurity rather than neuropsych dx; as you have suggested further on)

See answer to point 3.

10. Discussion (P14; L42): dealt not delt

This is changed in the text.

11. Discussion (P15; L22): bullied not bullyed

This is changed in the text.

12. Strengths and limitations: this is very limited. would be better to expand if possible.

This section was shortened during a previous revision due to word limitation, and is now somewhat expanded:

Old text:

Our registry data consists of a large natural population, containing all Norwegian citizens born from 1991 through 2012, with the possibility of long-term follow-up over several years.

We did not study comorbidity in particular. But as psychiatric comorbidity is common, one contact with health care could generate more than one diagnosis. Importantly, for the outcome any F diagnosis, there is only one contribution. Not all individuals have contributed with the same number of years, but all contributed years are complete, ensuring that data structure does not produce any biases for the relative age effects.

New text:

Our registry data consists of a large natural population, containing all Norwegian citizens born from 1991 through 2012, with the possibility of long-term follow-up over several years.

We did not study comorbidity in particular. But as psychiatric comorbidity is common, one contact with health care could generate more than one diagnosis. Also, our data do not distinguish between diagnoses given in psychiatric and somatic health care, or between main and secondary diagnoses. Importantly, for the outcome any F diagnosis, there is only one contribution. Not all individuals have contributed with the same number of years, but all contributed years are complete, ensuring that data structure does not produce any biases for the relative age effects.

Otherwise excellent and thought provoking!

Reviewer: 2
Dr. Linda Tran, Rivendell Child and Adolescent Unit
Comments to the Author
Your study utilizes a large population sample which provides important insights into long-term trajectories and outcomes based on the relative age paradigm. The amplification of the vulnerability for the relative younger children compared to their peers is both theoretically robust and practical e.g. regarding when to commence school or repeating a child at school etc. You take into consideration the effects of prematurity, gender and relative age in regard to "psychiatric issues". However, it is not a clear paper and the focus needs to be sharpened to bring out the points.

Specifics Suggestions:

Page 3, L 40: punctuation- full stop AFTER reference

This is changed in the text.

P3, L56: more details on what is meant that there is a collaboration with families? Does this mean they had any input e.g in study design ?

Our study group collaborates with "Prematurforeningen", the Norwegian patient association for families and individuals affected by preterm birth and possess valuable knowledge about their areas of focus. However, they have not had any direct influence on this project.

This is expanded in the text.

Old text:

Our project group collaborates with the organization for families and individuals affected by preterm birth.

New text:

Our project group collaborates with users (Home - Prematurforeningen) that emphasizes needs for more knowledge on the transition from pre-school to school for preterm born, however, they have not had any direct influence on analyses or manuscript in this project.

Page 4, L 35, Exposures: how did you arrive at these subgroups? It seems somewhat arbitrary. Why didn't you eg. use late preterm as a subgroup which is 34+0-36+6?

The subgroups were chosen as a pragmatic compromise between clinical and statistical issues. In Norway, the care services use 28, 32 and 37 weeks of gestation as cut-offs for eligibility to follow-up programs. Research findings from studies on preterm individuals provide substantial evidence of dose - response associations for many outcomes, with the highest risk for the most preterm individual. So ideally, a finer categorization of PT groups would be interesting. However, from a statistical point of view, there are issues of power and risk of chance findings with multiple testing. E.g subgroups consisting of only extremely and very preterm individuals are very small, especially with additional grouping based on birth month in the year. As the main aim of this analysis was to provide a supplemental evaluation of whether main findings differed according to degree of prematurity, we believe that the categories used are relevant (if not perfect).

Page 4, L 43: "In Norway, birth month is a good measure of relative age, since only about 0,6 % of Norwegian children delay starting school every year." – do you also mean that a school year in Norway starts in January of each year?

Thank you for this comment. We see that the description in the manuscript is unclear, and we have now changed the wording to improv clarity. The school year in Norway starts in august. All children who reach the age of six within a given calendar year commence school in August of that year, and almost all children follow their age cohort throughout primary schooling.

Old text:

Relative age was grouped according to birth month, categorized in four three-month groups (January-March, April-June, July-September and October-December). Individuals born in January-March were defined as the relatively oldest, and those born in October-December as the relatively youngest. In Norway, birth month is a good measure of relative age, since only about 0,6 % of Norwegian children delay starting school every year.

New text:

Relative age was grouped according to birth month, categorized in four three-month groups (January-March, April-June, July-September and October-December). In Norway, all children start school in August of the calendar year when they turn 6 years old, and only about 0,6 % of Norwegian children delay

starting school each year. Individuals born in January-March were defined as the relatively oldest, and those born in October-December as the relatively youngest.

Table 2:
- typo in eationg disorder

This is corrected (table S2)

- you refer to p values in the text but unsure where to find them in your data

In order to evaluate differences in relative age effects between groups (term vs preterm born, boys vs girls) we performed separate analyses including interaction terms (multiplicative associations) between these variables and birth month groups, at age 4-10 and 11-17 years. Presence of statistical interaction was evaluated systematically and reported whenever present, with p for interaction as a measure of the statistical strength of the group difference.

- if I understand the table correctly, I arrive at following conclusions:

o term boys have higher OR than preterm boys for all disorders except for ASD

o preterm girls have higher OR than term girls for all disorders except for ADHD and for ASD

o term boys and girls have higher OR for ADHD than their preterm counterparts

Table 2 shows relative age effects, and not prevalences. This means that the Odds Ratio shown represents how much more the relatively youngest children (born October-December) are diagnosed compared to the relatively oldest children (born January-March), within each group. Thus, estimates are presented contrasting relatively young and relatively old within each group and at each age: term boys and girls separately and within preterm boys and girls separately.

For example:

- Term boys have higher <u>relative age effects</u> than preterm boys for all disorders except for ASD = the difference in Odds between term boys born in October-December (relatively youngest) and term boys born in January-March (relatively oldest) is more pronounced than the same difference within preterm boys (relatively youngest vs relatively oldest).

- Preterm girls have greater <u>relative age effects</u> than term girls for all disorders except for ADHD and ASD.

- Term boys and girls have higher <u>relative age effects</u> for ADHD-diagnosis than their preterm counterparts

Page 6, L 5-14: What is an interaction term? A clearer explanation would be helpful

See above. We added the following in the statistics section:

In order to evaluate differences in relative age effects between groups (term vs preterm born, boys vs girls) we performed separate analyses including interaction terms (multiplicative associations) between these variables and birth month groups, at age 4-10 and 11-17 years. Presence of statistical interaction was evaluated systematically and reported whenever present, with p for interaction as a measure of the statistical strength of the group difference.

Page 7, L10: "Table S3 displays the subpopulation of 530427 individuals born in January-March and October-December." – do these 2 groups differ from the April – September groups?

Table 1 shows that the birth month groups throughout the year are similar and comparable regarding sociodemographic characteristics. The purpose of table S3 is to show that preterm and term individuals born October-December and preterm and term individuals born January-March do not differ substantially regarding sociodemographic characteristics, since relative age effect are compared within these groups.

This is now included in the text (Results, line 3):

Table 1 shows that sociodemographic and perinatal characteristics were fairly similar distributed between the four birth month groups.

Page 12, L 53: "The prevalence of healthcare use with psychiatric diagnoses was higher in preterm born compared to their term counterparts"- term boys have higher OR than preterm boys for all disorders except for ASD

This sentence highlights the prevalence within the entire preterm population vs. the entire term population (best seen in figure 2, which shows the prevalence/ absolute numbers), - and not relative age effects, ie. comparison based on birth month in the year (late/ early) within the groups, shown in table 2.

Page 13, L5: "Preterm children and boys, in particular, were more often registered with these (neuropsychiatric) conditions "– from table 2 it seems that term boys have higher OR than preterm boys for all disorders except for ASD

As above, this sentence highlights the prevalence within the entire preterm population and within boys, best shown in figure 3.

This is in contrast to relative age effects, which were stronger for girls than boys in several categories (here: neuropsychiatric conditions), as the second part of the sentence (.."but relative age effects were stronger for girls than boys in several categories.") highlights – shown in table 2.

Page 13, L42, typo: delt-dealt

This is changed in the text.

Page 13, L 45: "prescription of psychostimulants across adolescence for preterm girls"- is this trying to explain that the OR for ADHD is lower aged 11-17 compared to 4-10 in girls? But this reduction in OR is

for both genders and for term and preterm. Are there alternate explanations e.g. less ADHD symptoms due to maturity or e.g. kids leaving school earlier etc.?

This is trying to explain finding from our last study, where there were greater relative age effects (for use of psychostimulants) among preterm girls than preterm boys through adolescence (10-23 years), and that the present finding show the same tendency with greater relative age effects for preterm girls than preterm boys concerning some of the neuropsychiatric conditions.

P13, L 51: “boys are overdiagnosed with ADHD”- how do you explain that in table 2 girls (term and preterm) have higher OR for ADHD than boys for age 4-17 ?

Table 2 shows relative age effects and not prevalences, ie. OR of 1,53 for preterm girls 4-10 years means that the preterm girls born October-December have increased odds of 53% for a diagnosis of ADHD compared to preterm girls born January-March. The prevalences for the different conditions within the different groups is best shown I figure 2 and 3.

P14, L16: punctuation- full stop AFTER reference

This is changed in the text.

P14, L22: typo “bullyed”- bullied

This is changed in the text.

P14, L 22: “Furthermore, it appears that preterm children lack resilience to manage psychosocial stressors, which can result in a higher likelihood of experiencing adverse mental health outcomes.” – Table 2 seems to show the contrary; that term boys have higher OR than preterm boys for all disorders except for ASD and term children have higher OR for ADHD than their preterm counterparts

This sentence refers to the findings (that preterm children lack resilience to manage psychosocial stressors) presented in the cited reference. We have incorporated it as part of the explanation suggesting that preterm girls appear to be particularly vulnerable compared to other groups. The estimates presented in table 2 shows relative age effects.

P 14, L 25: “This may provide possible explanations for the observed risk in preterm girls relatively young.” – unsure if this sentence is complete? Or do you mean something like ‘preterm girls who are relatively younger than their peers’?

The latter represents the correct understanding, this is changed in the text as you suggest.

This paper would be considerably enhanced by answering the following questions:
1. Would you advise, based on this data, that premature boys be started at school later in the year or the following year than term boys?
2. Would you advise, based on this data, that premature girls be started at school later in the year or the following year than term girls?
3. Are preterm girls more vulnerable to a lower and neurodevelopmentally complicated trajectory than boys?
4. Is the relationship between psychiatric disorder and neurodevelopmental disorder categorical or dimension ie two aspects of the same vulnerability or at extremes are they different in kind?
5. Does prematurity confer any advantage or relative advantage on any subgroup?

Thanks for constructive feedback. We have revised the concluding remark to suggest something about these points. As for point 3, our findings suggest that preterm girls are particularly susceptible for relative age effects concerning mental health, and this is now highlighted in the conclusion.

According to our findings, prematurity was no advantage for any of the groups.

Point 4 is relevant, but in our opinion beyond the scope of this paper.

This paper now needs minor revisions to sharpen the message. I am happy to review the revised version and hope the comments are helpful.

VERSION 2 – REVIEW

**REVIEWER NAME**	***Shankari Arunanthy***
**REVIEWER AFFILIATION**	Royal Prince Alfred Hospital
**REVIEWER CONFLICT OF INTEREST**	N/A
**DATE REVIEW RETURNED**	11-Mar-2025

**GENERAL COMMENTS**	I really enjoyed this paper. Your response to previous concerns has been thorough and the paper reads quite clearly now. Congratulations!

**REVIEWER NAME**	***Linda Tran***
**REVIEWER AFFILIATION**	Rivendell Child and Adolescent Unit
**REVIEWER CONFLICT OF INTEREST**	N/A
**DATE REVIEW RETURNED**	09-Mar-2025

**GENERAL COMMENTS**	Thank you for making the changes.
